# Insight into the Systematics of Microfungi Colonizing Dead Woody Twigs of *Dodonaea viscosa* in Honghe (China)

**DOI:** 10.3390/jof7030180

**Published:** 2021-03-03

**Authors:** Dhanushka N. Wanasinghe, Peter E. Mortimer, Jianchu Xu

**Affiliations:** 1 CAS Key Laboratory for Plant Biodiversity and Biogeography of East Asia (KLPB), Kunming Institute of Botany, Chinese Academy of Science, Kunming 650201, Yunnan, China; dnadeeshan@gmail.com; 2 World Agroforestry, East and Central Asia, 132 Lanhei Road, Kunming 650201, Yunnan, China; 3 Honghe Center for Mountain Futures, Kunming Institute of Botany, Honghe County 654400, Yunnan, China

**Keywords:** Ascomycota, Asexual morph, Capnodiales, Greater Mekong Subregion, Hysteriales, Pleosporales, Sexual morph, Yunnan

## Abstract

Members of *Dodonaea* are broadly distributed across subtropical and tropical areas of southwest and southern China. This host provides multiple substrates that can be richly colonized by numerous undescribed fungal species. There is a severe lack of microfungal studies on *Dodonaea* in China, and consequently, the diversity, phylogeny and taxonomy of these microorganisms are all largely unknown. This paper presents two new genera and four new species in three orders of Dothideomycetes gathered from dead twigs of *Dodonaea viscosa* in Honghe, China. All new collections were made within a selected area in Honghe from a single *Dodonaea* sp. This suggests high fungal diversity in the region and the existence of numerous species awaiting discovery. Multiple gene sequences (non-translated loci and protein-coding regions) were analysed with maximum likelihood and Bayesian analyses. Results from the phylogenetic analyses supported placing *Haniomyces dodonaeae* gen. et sp. in the Teratosphaeriaceae family. Analysis of *Rhytidhysteron* sequences resulted in *Rhytidhysteron hongheense* sp. nov., while analysed Lophiostomataceae sequences revealed *Lophiomurispora hongheensis* gen. et sp. nov. Finally, phylogeny based on a combined dataset of pyrenochaeta-like sequences demonstrates strong statistical support for placing *Quixadomyces* *hongheensis* sp. nov. in Parapyrenochaetaceae. Morphological and updated phylogenetic circumscriptions of the new discoveries are also discussed.

## 1. Introduction

Fungi are cosmopolitan, featuring a broad geographic distribution and high level of diversity compared to plants and other organisms [1]. 140,000 fungal species have been listed in Kirk [2], and one recent overview of global fungi and fungus-like taxa by Wijayawardene et al. [3] listed approximately 100,000 known taxa. However, both numbers represent less than 5% of global fungal estimates [4,5]. There is a need to bridge the gap between our understanding of these missing fungi and their diversity. Numerous diverse habitats and substrates remain unexplored. It has also been observed that several countries and regions are bountiful repositories of many missing fungi, such as northern Thailand [6]. Despite this, fungi in Asia are relatively understudied [5]. Even though the Greater Mekong Subregion (GMS) hosts a high level of biodiversity and forms an integral part of the Indo-Burma Biodiversity Hotspot, fungi from this region largely remain a mystery. Yunnan Province, China, as part of the GMS, is home to an extremely wide variety of ecosystems. Mycologists working in Yunnan have recently focused their attention on abundant “less-researched habitats” for fungal occurrences, including caves, forests, grasslands, lakes, karst landscapes and mountains; accordingly, there is a rich body of literature documenting novel discoveries across the region [7,8,9,10,11,12,13,14,15,16,17,18,19].

The Honghe Hani and Yi Autonomous Prefecture is in south-eastern Yunnan Province. The region features a mountainous topography, numerous limestone deposits and a south-eastward decreasing elevation gradient. Owing to its abundant precipitation and heat as well as its dramatic altitudinal range and varied flora, this region harbours a rich diversity of plant species [20,21]. Along the altitudinal gradient, vegetation from lower to higher elevations range from tropical and montane rain forests to monsoon evergreen, montane mossy evergreen and summit mossy evergreen broad-leaved forests [22]. This complex topography and climatic diversity are both significant contributors to local biodiversity richness [23]. Among publications documenting fungal encounters across Yunnan Province, ascomycetes are critically neglected when compared to the amount of research on basidiomycetes [24]. Regrettably, studies on microfungi in Honghe are virtually non-existent. Except Marasinghe et al. [25], we could not find a single detailed account of microfungi in Honghe based on both morphological and phylogenetic analyses.

*Dodonaea viscosa* is a perennial evergreen woody shrub belonging to the family Sapindaceae. It is drought- and pollution-resistant as well as capable of growing on poor soils and rocky sites. The plant can also easily inhabit open areas and secondary forests [26,27]. A fast-growing plant, it typically grows 1 to 3 m in height but on rare occasions can reach up to 8 m [28]. *Dodonaea viscosa* is believed to have originated from Australia [29], though it grows throughout tropical and subtropical countries, including the African, Asian, Northern American and Southern American continents [30,31,32]. *Dodonaea viscosa* is effective at performing sand dune fixation and controlling coastal erosion since its roots function as excellent soil binders [33]. It can also be used to reclaim marshes. It is also grown as an ornamental plant owing to its shiny foliage and pink–red winged fruit [33]. Moreover, it is a well-known topic in environmental impact studies to determine the growth and yield of crops based on the presence of *D. viscosa* [27,34] as well as study its capacity to increase resilience to pollution [35,36] and drought [37]. In traditional medicine systems, plant parts such as the stem, leaves, seeds, roots, bark and aerial parts are used for various treatments [38]. Hossain [39] reported that extract obtained from *D. viscosa* has shown significant antidiabetic, antimicrobial, insecticidal, antioxidant, cytotoxic, antifertility, anti-inflammatory, analgesic, anti-ulcer, antispasmodic, anti-diarrheal and detoxification properties [27].

This study is the second in a series comprising an exhaustive taxonomic effort to document the microfungi of Yunnan Province [24]. In this study, we collected fresh fungal specimens from dead woody twigs of *Dodonaea* species at the Centre for Mountain Futures (CMF), an applied research centre jointly managed by World Agroforestry (ICRAF) and the Kunming Institute of Botany, Chinese Academy of Sciences (CAS), in Honghe County of the Honghe Hani and Yi Autonomous Prefecture. Using morphology and multi-gene phylogenetic evidence retrieved from the gathered ascomycetes, we characterized two new genera and four new species in the orders Capnodiales, Hysteriales and Pleosporales from dead twigs of *Dodonaea viscosa* in Honghe.

## 2. Materials and Methods

### 2.1. Herbarium Material and Fungal Strains

Fresh fungal materials were gathered from dead twigs of *Dodonaea viscosa* at CMF in Honghe County (Yunnan Province, China UTM/WGS84: 48 Q 216849–217075 E, 2592645–2592856 N, 600–750 m above sea level) during the dry season (April 2020). The local environment is characterized by poor eroded soils, steep valleys and a subtropical monsoon climate. Specimens were transported to the laboratory in Ziploc bags. Single spore isolation was conducted in accordance with methods described in Wanasinghe et al. [40]. Germinated spores were individually placed on potato dextrose agar (PDA) plates and grown at 20 °C in daylight. Dry herbarium materials were stored in the herbarium of Cryptogams Kunming Institute of Botany, Academia Sinica (KUN-HKAS). Living cultures were deposited at the Kunming Institute of Botany Culture Collection (KUMCC), Kunming, China and duplicated at China General Microbiological Culture Collection Centre (CGMCC). MycoBank numbers were registered as outlined in MycoBank (http://www.MycoBank.org accessed on 11 November 2020).

### 2.2. Morphological Observations

The morphology of external and internal macro-/micro-structures were observed as described in Wanasinghe et al. [24]. Images were captured with a Canon EOS 600D digital camera fitted to a Nikon ECLIPSE Ni compound microscope. Measurements were made with the Tarosoft (R) Image Frame Work program, and images used for figures were processed with Adobe Photoshop CS5 Extended version 10.0 software (Adobe Systems, San José, CA, USA).

### 2.3. DNA Extraction, PCR Amplifications and Sequencing

The extraction of genomic DNA was performed in accordance with the methods of Wanasinghe et al. [24], using the Biospin Fungus Genomic DNA Extraction Kit-BSC14S1 (BioFlux, P.R. China) following the instructions of the manufacturer. The reference DNA for the polymerase chain reaction (PCR) was stored at 4 °C for regular use and duplicated at −20 °C for long-term storage. The primers and protocols used for the amplification are summarized in Table 1. The amplified PCR fragments were then sent to a private company for sequencing (BGI, Ltd. Shenzhen, P.R. China).

### 2.4. Molecular Phylogenetic Analyses

#### 2.4.1. Sequence Alignment

Sequences featuring a high degree of similarity were determined from a BLAST search to identify the closest matches with taxa in Dothideomycetes and recently published data [49,53,54,55,56]. Initial alignments of the acquired sequence data were first completed using MAFFT v. 7 (http://mafft.cbrc.jp/alignment/server/index.html accessed on 18 January 2021) [57,58] and manually clarified in BioEdit v. 7.0.5.2 when indicated [59].

#### 2.4.2. Phylogenetic Analyses

Single-locus data sets were scanned for topological incongruences between loci for members of the analyses. Conflict-free alignments were concatenated into a multi-locus alignment that underwent maximum-likelihood (ML) and Bayesian (BI) phylogenetic analyses. Evolutionary models for BI and ML were selected independently for every locus using MrModeltest v. 2.3 [60] under the Akaike Information Criterion (AIC) implemented in PAUP v. 4.0b10.

The CIPRES Science Gateway platform [61] was used to perform RAxML and Bayesian analyses. ML analyses were made with RAxML-HPC2 on XSEDE v. 8.2.10 [62] employing the GTR+GAMMA swap model with 1000 bootstrap repetitions.

MrBayes analyses were performed setting GTR+I+GAMMA for 2–5 million generations, sampling every 100 generations and ending the run automatically when standard deviation of split frequencies dropped below 0.01 with a burnin fraction of 0.25. ML bootstrap values equal or greater than 60% and Bayesian posterior probabilities (BYPPs) greater than 0.95 were placed above each node of every tree.

Phylograms were visualized with FigTree v1.4.0 program [63] and reassembled in Microsoft PowerPoint (2007) and Adobe Illustrator^®^ CS5 (Version 15.0.0, Adobe^®^, San Jose, CA, USA). Finalized alignments and trees were deposited in TreeBASE, submission ID: S27699 (http://purl.org/phylo/treebase/phylows/study/TB2: S27699).

## 3. Results

### 3.1. Global Checklist of Fungi on Dodonaea Viscosa

Information for the global checklist (Table 2) was retrieved from the Agriculture Research Service Database generated by the United States Department of Agriculture (USDA) [64], related books and research papers. This checklist includes fungal species associated with *Dodonaea viscosa* and the countries from which they were recorded.

### 3.2. Phylogenetic Analyses

**Figure 1:**
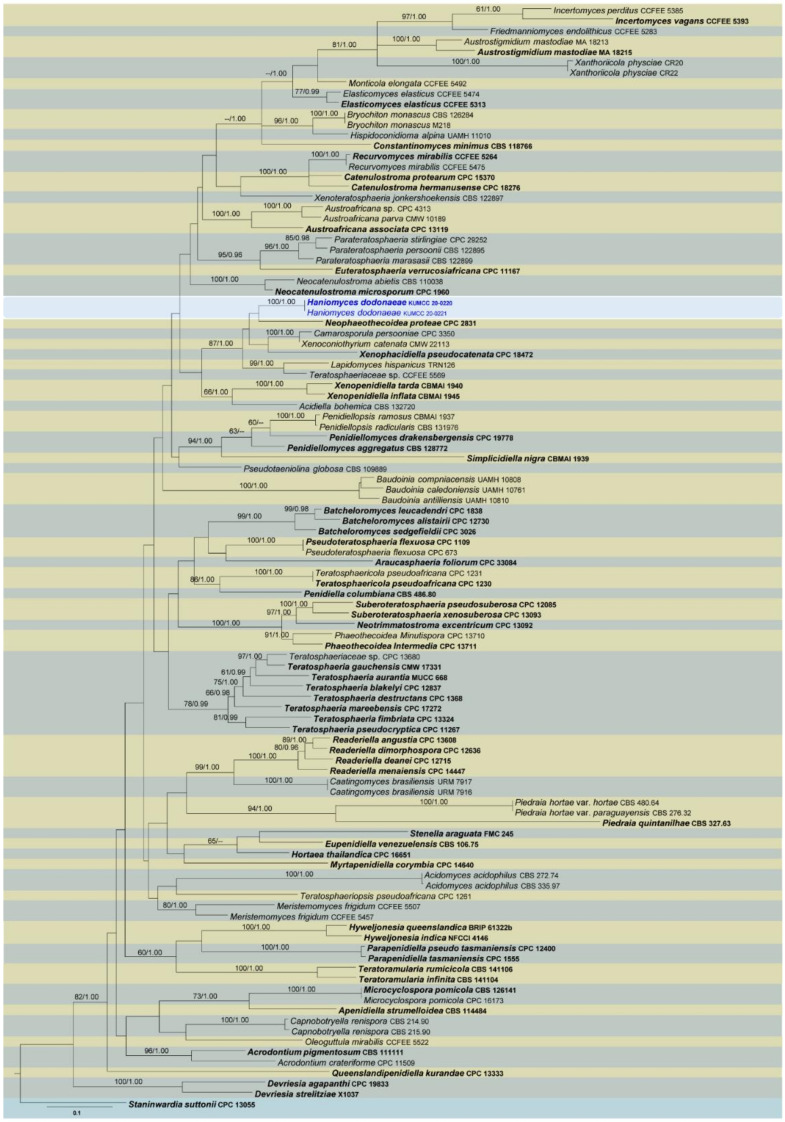
RAxML tree based on a combined dataset of partial LSU, ITS, *rpb*2, *act*, *cal* and *tef*1 DNA sequence analysis in Teratosphaeriaceae. The tree is rooted to *Staninwardia suttonii* (CPC 13055). Bootstrap support values for ML equal to or greater than 60%, Bayesian posterior probabilities (BYPP) equal to or greater than 0.95 are presented as ML/BI above nodes. Known genera are indicated with coloured blocks. Blue represents new isolates. The ex-type strains are indicated in **bold**. The scale bar presents the expected number of nucleotide substitutions per site.

**Figure 2:**
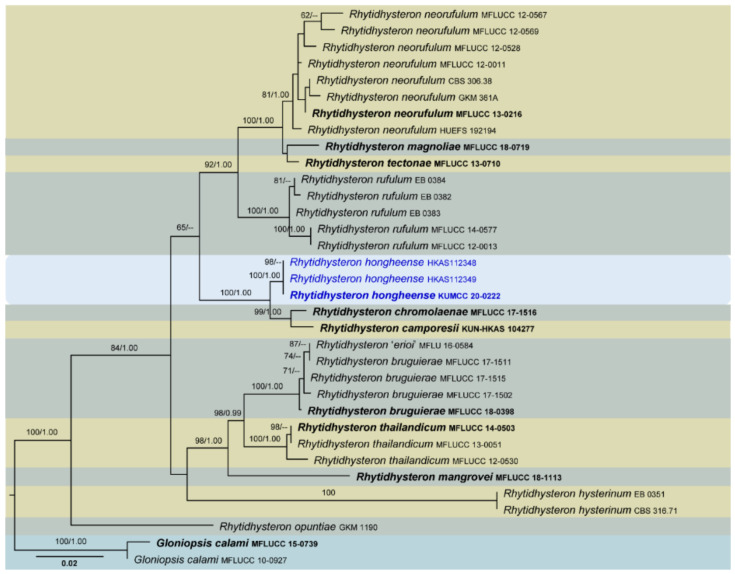
RAxML tree based on a combined dataset of partial SSU, LSU, ITS and *tef*1 DNA sequence analysis in *Rhytidhysteron*. The tree is rooted to *Gloniopsis calami* (MFLUCC 15-0739, MFLUCC 10-0927). Bootstrap support values for ML equal to or greater than 60% and BYPP equal to or greater than 0.95 are shown as ML/BI above the nodes. Known species are indicated with coloured blocks. Blue represents new isolates. The ex-type strains are indicated in **bold**. The scale bar represents the expected number of nucleotide substitutions per site.

**Figure 3:**
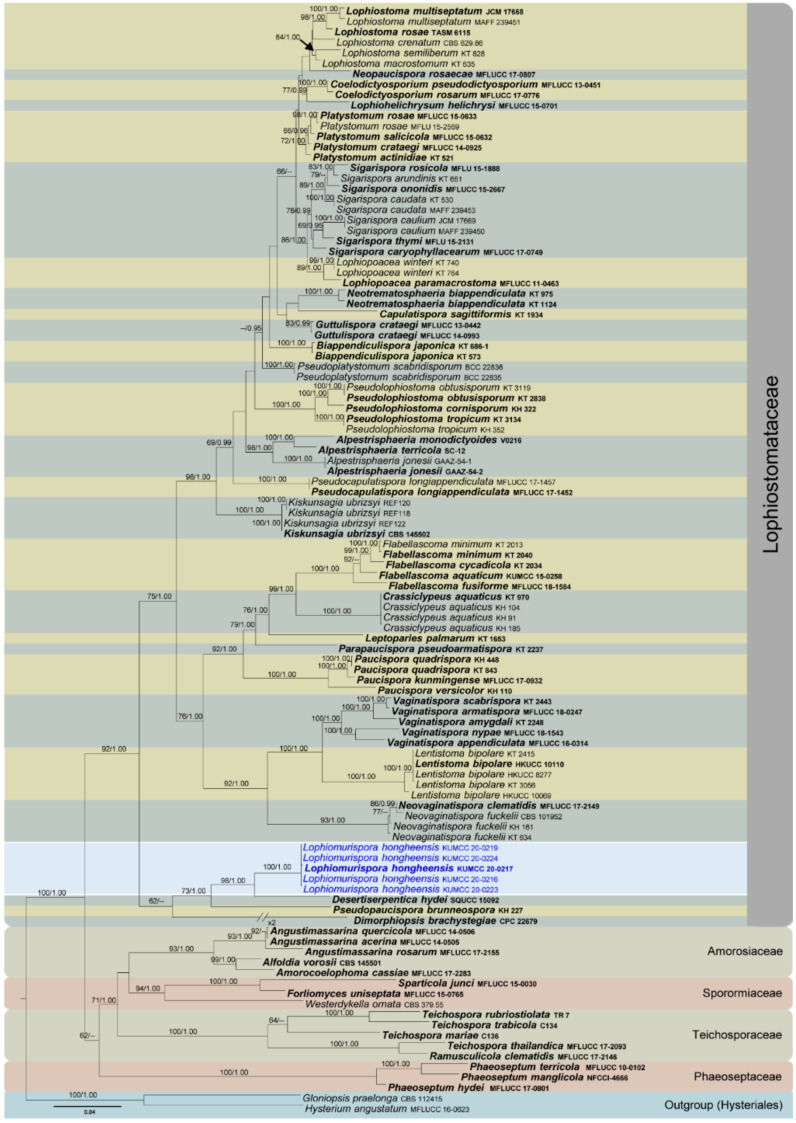
RAxML tree based on a combined dataset of partial SSU, LSU, ITS, *tef*1 and *rpb*2 DNA sequence analysis in Lophiostomataceae. The tree is rooted to *Gloniopsis praelonga* (CBS 112415) and *Hysterium angustatum* (MFLUCC 16-0623). Bootstrap support values for ML equal to or greater than 60% and BYPP equal to or greater than 0.95 are shown as ML/BI above the nodes. Known families and selected genera are indicated with coloured blocks. Blue represents new isolates. The ex-type strains are indicated in **bold**. The scale bar represents the expected number of nucleotide substitutions per site.

**Figure 4:**
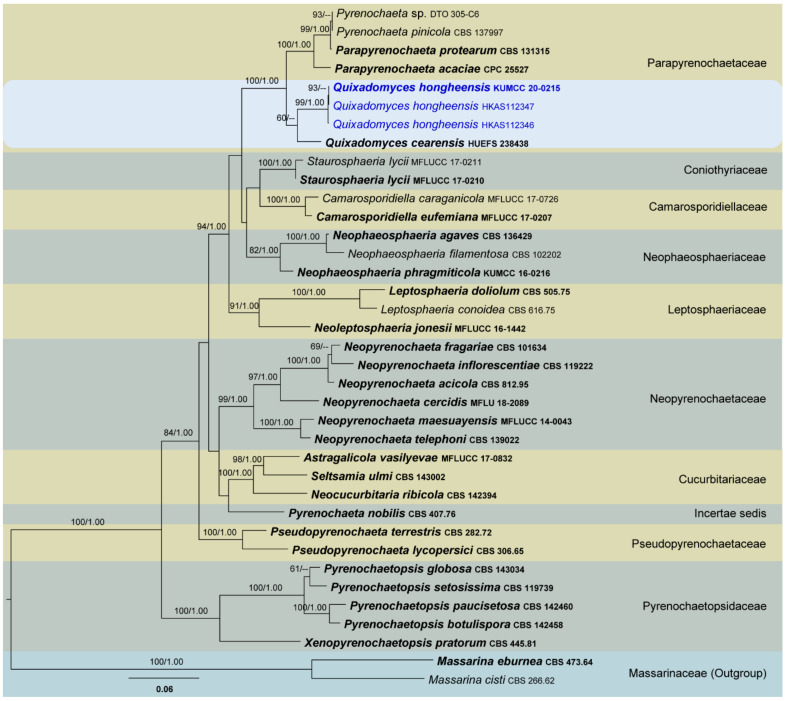
RAxML tree based on a combined dataset of partial LSU, SSU, ITS, *rpb*2, *tef*1 and *btub* DNA sequence analysis in Pleosporineae. The tree is rooted to *Massarina cisti* (CBS 266.62) and *M*. *eburnea* (CBS 473.64). Bootstrap support values for ML equal to or greater than 60% and BYPP equal to or greater than 0.95 are shown as ML/BI above the nodes. Known families and the genus *Quixadomyces* are indicated with coloured blocks. Blue represents new isolates. The ex-type strains are indicated in **bold**. The scale bar represents the expected number of nucleotide substitutions per site.

Four phylogenetic analyses were performed using the acquired sequences from GenBank (Table 3). The first is a phylogenetic overview of the genera treated in Teratosphaeriaceae (Figure 1), while the remaining three alignments represent the species in *Rhytidhysteron* (Figure 2), an overview of the phylogeny of the genera treated in Lophiostomataceae (Figure 3) and *Parapyrenochaeta*, and allied genera in Pleosporineae (Figure 4). Other details related to ML and BI analyses from different datasets are presented in Table 4. The acquired phylogenetic results are discussed where applicable in the notes below.

### 3.3. Taxonomy of Fungi Colonising Dodonaea Viscosa Twigs

In the current study, two new genera and four novel species were found. These taxa are subsequently described below.

Class Dothideomycetes O.E. Erikss. and Winka, Myconet 1: 5 (1997)

Capnodiales Woron., Annales Mycologici 23: 177 (1925)

Teratosphaeriaceae Crous and U. Braun, Studies in Mycology 58: 8 (2007)

***Haniomyces* J.C. Xu gen. nov.**

MycoBank: MB837991

*Etymology*: The generic epithet refers to the “Hani” ethnic group in Honghe County, Yunnan Province, China.

It is *saprobic* on dead twigs and branches in terrestrial habitats. Sexual morph: the *ascomata* is a scattered, immersed to semi-immersed, subglobose to conical or shaped irregularly, glabrous, brown to dark brown ostiolate. The *ostiole* is a short papillate, black, smooth periphysate. The *peridium* comprises cells of *textura angularis*. The *hamathecium* comprises numerous, filamentous, branched, septate, pseudoparaphyses. The *asci* are eight-spored, bitunicate, fissitunicate, clavate, with a pedicel, apically rounded with or without an ocular chamber. The *ascospores* overlap the biseriate, are ellipsoidal to sub-fusiform, hyaline, one-septate, with small to large guttules in each cell, with the ends remaining rounded, surrounded by a distinct mucilaginous sheath. Asexual morph: Coelomycetous. The *conidiomata* are sporodochial on PDA, globose, solitary or aggregated, semi-immersed, black, exuding yellow conidial masses. *Conidiophores* and conidiogenous cells were not observed in vitro. The *conidia* are solitary, aseptate, globose to ellipsoid, with the hyaline becoming medium to golden brown, and finely verruculose.

Type species: Haniomyces dodonaeae

**Figure 5:**
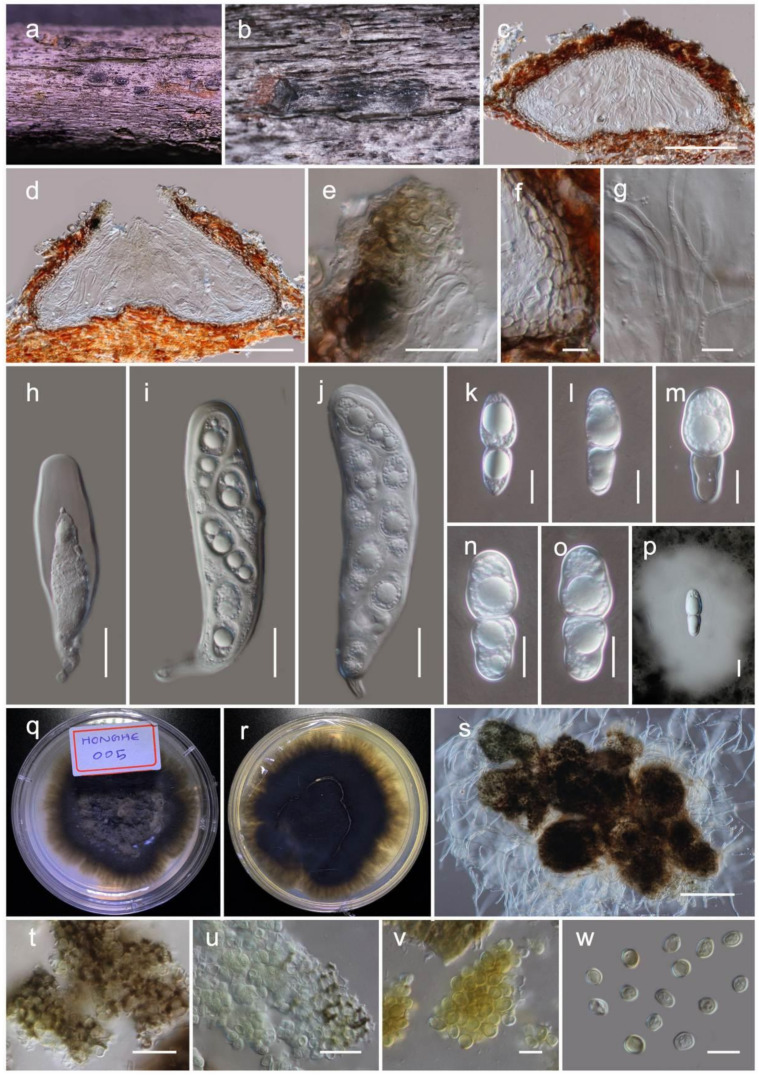
The sexual (HKAS110128, holotype) and asexual (KUMCC 20-0220, ex-type) morphs of *Haniomyces dodonaeae*. (**a,b**) ascomata on the dead woody twigs of *Dodonaea viscosa*; (**c,d**) vertical section of ascoma; (**e**) periphyses; (**f**) peridium; (**g**) pseudoparaphyses; (**h**–**j**) asci; (**k**–**p**) ascospores (**p** in Indian Ink); (**q,r**) colony on potato dextrose agar (PDA) (**r** from the bottom); (**s**) squashed pycnidia which were produced on PDA; (**t**) pycnidia wall; (**u**–**w**) conidia. Scale bars, (**c,d**) 100 µm; (**e,h**–**j,t,u**) 20 µm; (**f**,**k**–**p,v,w**) 10 µm; (**s**) 200 µm.

***Haniomyces dodonaeae* Wanas**. and Mortimer sp. nov. (Figure 5)

MycoBank: MB837997

*Etymology*: The specific epithet reflects the host genus *Dodonaea*.

Holotype: HKAS110128

It is *saprobic* on dead twigs of *Dodonaea viscosa* Jacq. (Sapindaceae). Sexual morph: the *ascomata* is a 150–200 μm high, 350–450 μm diam. (M = 165.4 × 390.3 µm, *n* = 5), scattered, semi-immersed to erumpent, subglobose to conical or shaped irregularly, flattened base, glabrous, brown to dark brown ostiolate, fused with host tissues. The *ostiole* is a short papillate, black and smooth, with hyaline periphyses (15–25 μm long, 1.5–2 μm wide). The *peridium* 5–10 µm wide at the base, 10–20 µm wide at sides, comprising 2–4 layers, outer layer pigmented, comprising reddish brown to dark brown, with thin-walled cells of *textura angularis*, and an inner layer composed of hyaline, loosen, cells of *textura angularis*. The *hamathecium* comprises numerous, 2–3 µm wide, filamentous, branched, septate, pseudoparaphyses. The *asci* are 110–130 × 25–35 µm (M = 118.5 × 31.2 µm, *n* = 20), eight-spored, bitunicate, fissitunicate, clavate, with a short pedicel (10–15 μm long), apically rounded with an ocular chamber. The *ascospores* 25–35 × 12–15 µm (M = 32.2 × 14.3 µm, *n* = 30), overlap the biseriate, are ellipsoidal to sub-fusiform, hyaline, one-septate, with the septum almost median, deeply constricted at the middle septum, with the upper cell wider than the lower cell, and are smooth-walled with small to large guttules in each cell, rounded at both ends and covered by a distinct mucilaginous sheath (30–50 µm, diam.). Asexual morph: Coelomycetous. The *conidiomata* are up to 250 μm diam., sporodochial on PDA, globose, solitary or aggregated, semi-immersed, black, exuding yellow conidial masses. *Conidiophores* and conidiogenous cells were not observed in vitro. The *conidia* are 5.5–7.5 × 4.5–5.5 µm (M = 6.4 × 5.4 µm, *n* = 30), solitary, aseptate, globose or ellipsoid, with the hyaline becoming medium to golden brown, and finely verruculose.

Culture characteristics: the colonies on PDA reached a 3 cm diameter after 2 weeks at 20 °C. They were circular has a serrate margin, whitish at the beginning, becoming brown at the centre and brownish green towards the margin after 4 weeks. They were slightly raised, and reverse blackish brown. The hyphae septate were branched, hyaline, thin, and smooth-walled.

Known distribution: Yunnan, China, on *Dodonaea viscosa*.

Material examined: China, Yunnan, Honghe Hani and Yi Autonomous Prefecture, Honghe County, 23.421068 N, 102.229128 E, 735 m, on dead twigs of *Dodonaea viscosa*, 22 April 2020, D.N. Wanasinghe, Honghe 005 (HKAS110128, holotype), ex-type living culture, KUMCC 20-0220, *ibid*. 23.419206 N, 102.231375 E, 618 m, Honghe 010 (HKAS110125, paratype), ex-paratype living culture, KUMCC 20-0221.

Hysteriales Lindau, Die Natürlichen Pflanzenfamilien nebst ihren Gattungen und wichtigeren Arten 1 (1): 265 (1897

Hysteriaceae Chevall., Flore Générale des Environs de Paris 1: 432 (1826)

*Rhytidhysteron* Speg., Anales de la Sociedad Científica Argentina 12 (4): 188 (1881)

**Figure 6:**
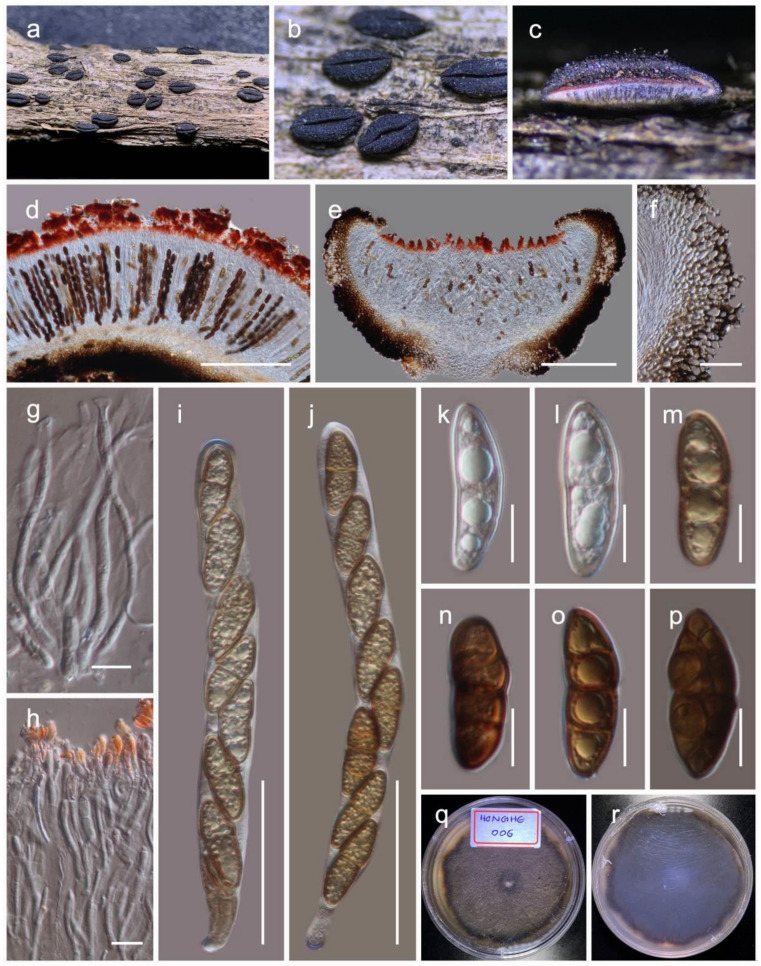
*Rhytidhysteron hongheensis* (HKAS110133, holotype). (**a,b**) Appearance of hysterothecia on the dead woody twigs of *Dodonaea viscosa*; (**c,d**) horizontal section of hysteriothecium; (**e**) vertical section of hysteriothecium; (**f**) cells of peridium; (**g,h**) pseudoparaphyses; (**i,j**) asci; (**k**–**p**) ascospores; (**q,r**) colony on PDA (**r** from the bottom). Scale bars, (**d,e**) 200 µm; (**f,i,j**) 50 µm; (**g,h**,**k**–**p**) 10 µm.

***Rhytidhysteron hongheense*****Wanas. sp. nov.** (Figure 6)

MycoBank: MB837992

*Etymology*: The specific epithet is derived from Honghe County, Yunnan Province, China.

Holotype: HKAS110133

It is *aaprobic* on dead twigs of *Dodonaea* Mill. (Sapindaceae). Sexual morph: The *hystherothecia* is 1200–2000 μm long × 350–500 high × 600–1000 µm diam. (M = 1590 × 410 × 840 µm, *n* = 10), arising singly or in small groups, sessile, and slightly erumpent from the substrate. The *receptacle* is cupulate, black, flat or slightly concave, with a slightly dentate margin. The *excipulum* are 70–100 µm wide, with the ectal excipulum narrow layered, deep, and thick-walled, with black cells of *textura globulosa* to *textura angularis*; the medullary excipulum is composed of narrow, long, thin-walled, hyaline to brown cells of *textura angularis*. The *hamathecium* are 2.5–4 µm wide, numerous, propoloid, pseudoparaphyses, exceeding asci in length, apically swollen, branched and reddish-orange pigmented. The branched apices form a layer on hymenium to develop pseudo-epithecium. The *asci* are 140–180 × 12–16 µm (M = 163.3 × 13.8 µm, *n* = 20), eight-spored, long cylindrical, short pedicellate, and is rounded at apex. The *ascospores* 20–33 × 9–13 µm (M = 28.2 × 11.2 µm, *n* = 30), overlap the uniseriate, are hyaline to light brown, one-septate, with wrinkled walls when young, becoming dark brown at maturity. They are ellipsoid with conical ends, regularly three-septate, and rarely muriform with one longitudinal septum, smooth walled, guttulate. Asexual morph: Undetermined.

Culture characteristics: Colonies on PDA reached a 4 cm diameter after 2 weeks at 20 °C. The colony was dense, circular, slightly raised, and the surface was smooth, with an undulated edge, with floccose which were greenish grey at the centre and brown towards margin from the top and reverse dark brown. The hyphae septate were branched, hyaline, thin, and smooth-walled.

Known distribution: Yunnan, China, on *Dodonaea*.

Material examined: China, Yunnan, Honghe Hani and Yi Autonomous Prefecture, Honghe County, 23.421068 N, 102.229128 E, 735 m, on dead twigs of *Dodonaea*, 22 April 2020, D.N. Wanasinghe, Honghe 006 (HKAS110133, holotype), ex-type culture, KUMCC 20-0222. *ibid*. on dead twigs of *Dodonaea viscosa*, 08 December 2020, DWH6-1 (HKAS112348). *ibid*. 07 December 2020, DWH7-2 (HKAS112349).

Pleosporales Luttr. ex M.E. Barr, Prodromus to class Loculoascomycetes: 67 (1987)

Lophiostomataceae Sacc., Sylloge Fungorum 2: 672 (1883)

***Lophiomurispora*** Wanas. and Mortimer, gen. nov.

MycoBank: MB837993

*Etymology*: The generic epithet stems from the combined two words ‘‘lophio’’ and ‘‘murispora’’, referring to muriform ascospores in Lophiostomataceae.

It is *saprobic* on woody substrates in terrestrial habitats. Sexual morph: The *ascomata* is a solitary or gregarious, semi-immersed, erumpent through the host surface, coriaceous to carbonaceous, dark brown to black, globose to subglobose or conical ostiolate. The *ostiole* is a slit-like, central papillate, with or without a crest, opening by an apical, lysigenous pore or dehiscence, comprising hyaline periphyses or hyaline to lightly pigmented, pseudoparenchymatous cells. The *peridium* is broad at the apex and thinner at the base, comprising two strata with several layers of brown or lightly pigmented to hyaline cells of *textura angularis* to *textura prismatica*, fusing and indistinguishable from the host tissues. The *hamathecium* comprises many branched, septate, cellular pseudoparaphyses, located between and above the asci, embedded in a gelatinous matrix. The *asci* are eight-spored, bitunicate, fissitunicate, cylindric-clavate, pedicellate, and apically rounded, with an ocular chamber. The *ascospores* are uni- to bi-seriate, partially overlapping, and are hyaline when immature, becoming brown to dark brown when mature. They are ellipsoidal to fusiform, muriform, two-to-eight-transversely septate, with one-to-two-longitudinal septa, constricted at the central septum, with or without a mucilaginous sheath. Asexual morph: Coelomycetous. The *conidiomata* is pycnidial, phoma-like, solitary, gregarious, dark brown to black, immersed or slightly erumpent, coriaceous to carbonaceous, papillate or apapillate. The *conidiomata wall is* multi-layered, with three to four outer layers of brown-walled pseudoparenchymatous cells, with the inner most layer being thin and hyaline. The *conidiophores* are long, septate, and sparsely branched, which are formed from the inner most layer of the pycnidium wall. The *conidiogenous cells* are phialidic, cylindrical, hyaline, flexuous and smooth, with a short collarette. The *conidia* are hyaline, aseptate, straight to curved, ellipsoidal with rounded ends, thin-walled, smooth, and numerous.

Type species: Lophiomurispora hongheensis

**Figure 7:**
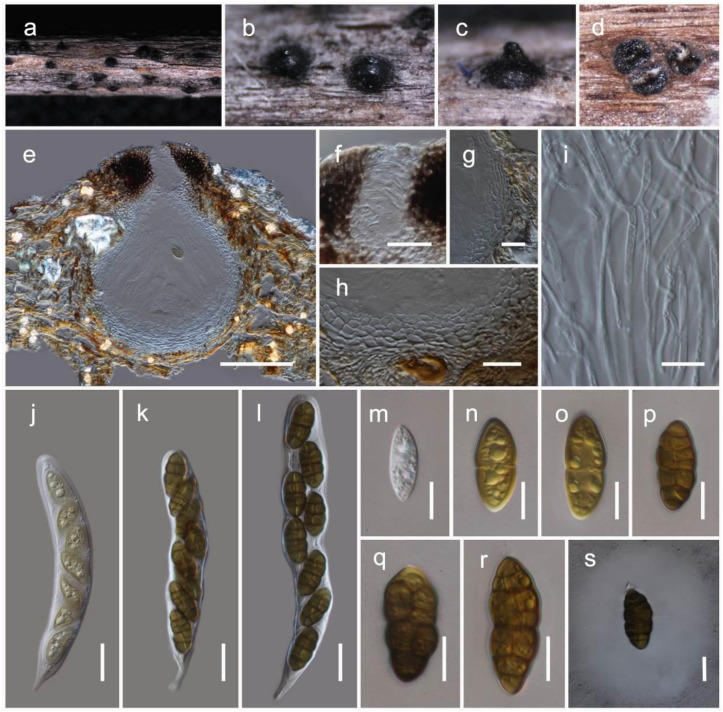
Sexual morph of *Lophiomurispora hongheensis* (HKAS110127, holotype). (**a**–**c**) Ascomata on the dead woody twigs of *Dodonaea viscosa*; (**d**) cross section of ascomata; (**e**) vertical section of ascoma; (**f**) closeup of ostiole; (**g,h**) peridium; (**i**) pseudoparaphyses; (**j**–**l**) asci; (**m**–**s**) ascospores (**s** in Indian Ink); Scale bars, (**e**) 100 µm; (**f**–**h,j**–**l**) 20 µm; (**i**,**m**–**s**) 10 µm.

**Figure 8:**
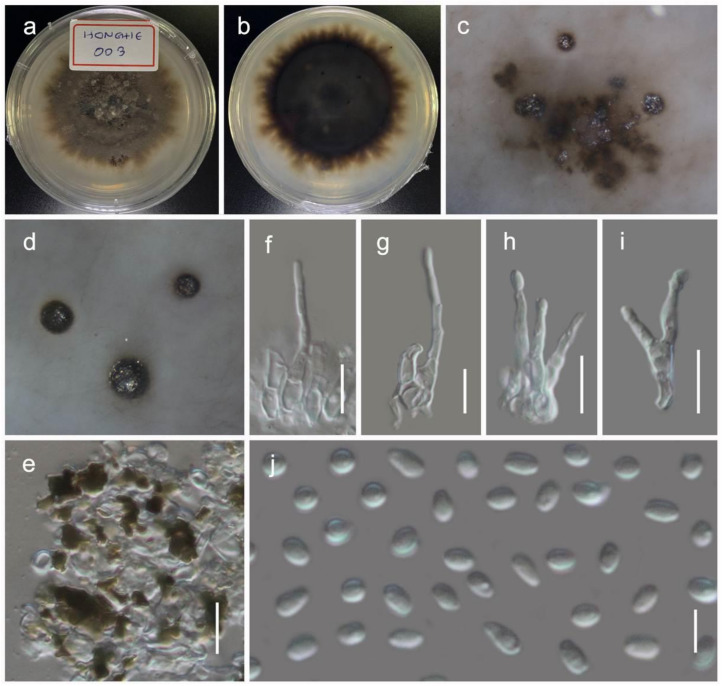
Asexual morph of *Lophiomurispora hongheensis* (KUMCC 20-0217, ex-type culture). (**a,b**) colony on PDA (**b** from the bottom); (**c,d**) immersed pycnidia in PDA (from the bottom); (**e**) pycnidia wall; (**f**–**i**) conidiophore; (**j**) conidia. Scale bars, (**e**–**i**) 10 µm; (**j**) 5 µm.

***Lophiomurispora hongheensis*** Wanas. sp. nov. (Figure 7 and Figure 8)

MycoBank: MB 837998

*Etymology*: The specific epithet is derived from Honghe County, the region of Yunnan Province in which this species was gathered.

Holotype: HKAS110127

It is *saprobic* on dead twigs of *Dodonaea viscosa* Jacq. (Sapindaceae) in terrestrial habitats. Sexual morph: The *ascomata* is a 280–360 μm high, 200–250 μm diam. (M = 318.6 × 232.7 µm, *n* = 5), scattered to gregarious, immersed, coriaceous, dark brown to black, globose to subglobose ostiolate. The *ostiole* is a 70–100 μm long, 40–80 μm diam. (M = 82.1 × 64.8 µm, *n* = 5), crest-like, central papillate, with a pore-like opening, comprising hyaline periphyses. The *peridium* is 20–30 μm wide at the base, 30–60 μm wide at the sides, broad at the apex, comprising two strata, with outer stratum composed of small, pale brown to brown, slightly flattened, thick-walled cells of *textura angularis*, fusing and indistinguishable from the host tissues. The inner stratum is composed of several layers with lightly pigmented to hyaline cells of *textura angularis* to *textura prismatica*. The *hamathecium* comprises 1–2 μm wide, branched, septate, cellular pseudoparaphyses, situated between and above the asci, embedded in a gelatinous matrix. The *asci* are 120–160 × 17–22 μm (M = 135.2 × 18.5 μm, *n* = 15), eight-spored, bitunicate, fissitunicate, cylindric-clavate, with a short pedicel, and is rounded at the apex, with an ocular chamber. The *ascospores* are 25–30 × 11–13 μm (M = 27.8 × 12 µm, *n* = 30), uni- to bi-seriate, overlapping, and are initially hyaline, turning brown at maturity. They are ellipsoidal to fusiform, muriform, four-to-eight-transversely septate, with one-to-two-longitudinal septa. They are slightly curved, deeply constricted at the central septum, slightly constricted at the remaining septa, conically rounded at the ends, and smooth-walled, with a distinct mucilaginous sheath. Asexual morph: Coelomycetous. The *conidiomata* is 1–1.5 mm diam. pycnidial, phoma-like, solitary, gregarious, dark brown to black, and immersed, with a sphaerical mass of slimy conidia oozing out at ostiolar apex. The *conidiomata wall* is multi-layered, with brown-walled pseudoparenchymatous cells, with a hyaline inner most layer. The *conidiophores* are 10–15 × 1.5–2.5 μm long (M = 12.4 × 2.1 µm, *n* = 15), septate and sparsely branched, which are formed from the inner most layer of the pycnidium wall. The *conidiogenous cells* are phialidic, cylindrical, hyaline, flexuous and smooth, with a short collarette. The *conidia* are 2.5–4 ×1.5–2 μm (M = 3 ×1.7 μm, *n* = 50), hyaline, aseptate, straight to curved, ellipsoidal with rounded ends, and are thin-walled, smooth-walled, and numerous.

Culture characteristics: the colonies on PDA reached a 4 cm diameter after 2 weeks at 20 °C. They were circular, had a serrate margin, and were whitish at the beginning, becoming greenish-brown 4 weeks later. They were slightly raised, and reverse dark brown. The hyphae septate were branched, hyaline, thin, and smooth-walled.

Known distribution: Yunnan, China, on *Dodonaea viscosa*.

Material examined: China, Yunnan, Honghe Hani and Yi Autonomous Prefecture, Honghe County, 23.421068 N, 102.229128 E, 735 m, on dead twigs of *Dodonaea viscosa*, 22 April 2020, D.N. Wanasinghe, Honghe 003 (HKAS110127, holotype), ex-type culture, KUMCC 20-0217, *ibid*. 23.419206 N, 102.231375 E, 618 m, Honghe 008 (HKAS110129, paratype), ex-paratype living culture, KUMCC 20-0223, *ibid*. 23 April 2020, *ibid*. DWHH07-1 (HKAS110130), living culture, KUMCC 20-0224, DWHH01 (HKAS110132), living culture, KUMCC 20-0216, *ibid*. DWHH04 3 (HKAS110131), living culture, KUMCC 20-0219.

Parapyrenochaetaceae Valenz-Lopez, Crous, Stchigel, Guarro and J.F. Cano, Studies in Mycology 90: 64 (2017)

*Quixadomyces* Cantillo and Gusmão, Persoonia 40: 317 (2018)

**Figure 9:**
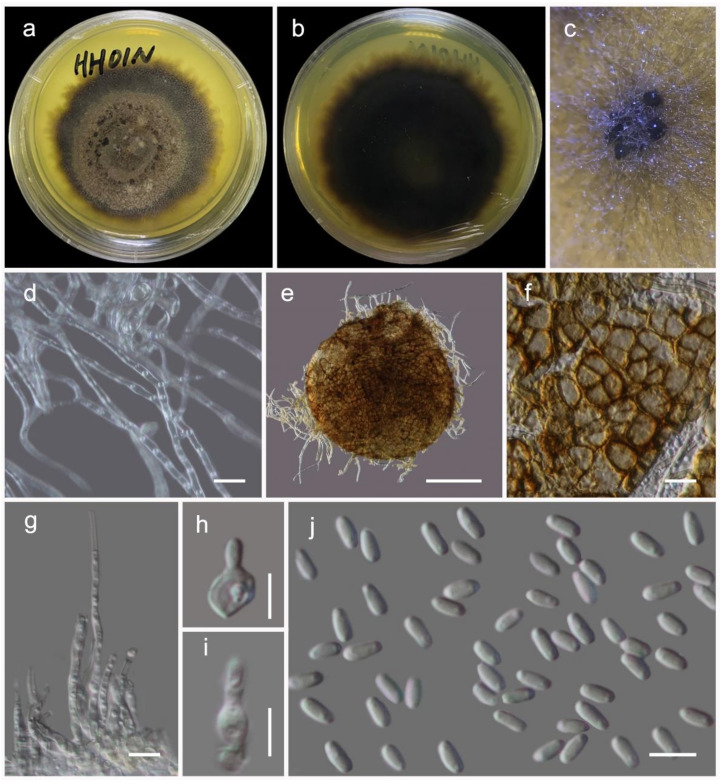
*Quixadomyces hongheensis* (KUMCC 20-0215, ex-type culture). (**a,b**) colony on PDA (**b** from the bottom); (**c**) pycnidia on PDA; (**d**) mycelia; (**e**) squashed pycnidia; (**f**) pycnidia wall; (**g**) paraphyses; (**h,i**) conidiophore; (**j**) conidia. Scale bars, (**d,f,g**) 10 µm; (**e**) 200 µm; (**h**–**j**) 5 µm.

***Quixadomyces hongheensis*** Wanas. sp. nov. (Figure 9)

MycoBank: MB837994

*Etymology*: The specific epithet is derived from Honghe County, Yunnan Province, China.

Holotype: HKAS110126

It is *saprobic* on dead twigs of *Dodonaea viscosa* Jacq. (Sapindaceae) in terrestrial habitats. Sexual morph: Undetermined. Asexual morph: Coelomycetous. The *conidiomata* is immersed to erumpent, solitary, globose, brown, from 200–300 μm diam, with a central ostiole, exuding a hyaline conidial mass. It has a wall of two to three layers of brown *textura angularis*. The *paraphyses* are 20–100 μm long, 2–3 μm wide, cylindrical, hyaline, septate, and smooth. The *conidiophores* are mostly reduced to conidiogenous cells. The *conidiogenous cells* are 5–8 × 3.5–5 μm (M = 6.4 × 3.1 µm, *n* = 15), lining the inner cavity, hyaline, smooth, are ampulliform to subcylindrical, and are phialidic with periclinal thickening. The *conidia* are 3–4.7 × 1.2–2 (M = 3.7 × 1.7 µm, *n* = 60) μm, solitary, hyaline, smooth, aseptate, and allantoid with obtuse ends.

Culture characteristics: The colonies on PDA reached a 4 cm diameter after 2 weeks at 20 °C. They were circular, had a serrate margin, and were greenish brown after 4 weeks. They were slightly raised, and reverse dark brown. The hyphae septate were branched, hyaline, thin, and smooth-walled.

Known distribution: Yunnan, China, on *Dodonaea viscosa*. 

Material examined: China, Yunnan, Honghe Hani and Yi Autonomous Prefecture, Honghe County, 23.421068 N, 102.229128 E, 735 m, on dead twigs of *Dodonaea viscosa*, 22 April 2020, D.N. Wanasinghe, Honghe 01-N (HKAS110126, holotype), ex-type living culture, KUMCC 20-0215. 08 December 2020, HDW4-1 (HKAS112347). *ibid*. HDW4-3 (HKAS112346).

## 4. Discussion

Teratosphaeriaceae was introduced by Crous et al. [187]. Given that it is composed of 61 genera, it is regarded as one of the largest families in Dothideomycetes [188]. Members of this family are adapted to a broad range of life modes and can be saprobic, plant and human pathogenic, rock-inhabiting and endophytic; accordingly, they are widely distributed across varied terrain [49,136,139,188,189]. We have included representative sequence data of all available genera listed in Hongsanan et al. [188] for the phylogenetic analyses (except *Davisoniella*, *Pachysacca* and *Placocrea*, which lack DNA-based sequence data). Among them, *Aulographina* was grouped in Venturiales, and *Leptomelanconium* was related to Helotiales in the initial analysis. Therefore, they were excluded from the final analysis (Figure 1). In addition, representative taxa for *Piedraia* were included in the final dataset that were phylogenetically closely related to Teratosphaeriaceae. However, this genus is still considered a member in Piedraiaceae. The phylogeny generated herein (Figure 1) is congruent with those of other published studies to resolve intergeneric relationships in Teratosphaeriaceae [49,188]. In the combined LSU, ITS, *rpb*2, *act*, *cal* and *tef*1 data analysis, 58 clades are recognized from the ingroup taxa. Two strains from our new collections constitute a distinct monophyletic lineage (subclade 17, Figure 1) within the genera in Teratosphaeriaceae, which we introduce as a new genus.

The phylogeny (Figure 1) reveals a close relationship between two strains of the newly collected fungus (*Haniomyces dodonaeae*) to *Camarosporula persooniae*, *Lapidomyces hispanicus*, *Neophaeothecoidea proteae*, Teratosphaeriaceae sp. (CCFEE 5569), *Xenoconiothyrium catenata* and *Xenophacidiella pseudocatenata*, with 87% ML and 1.00 BYPP support values. Among them, only *Camarosporula persooniae* is reported from the sexual morph, and despite the high degree of phylogenetic similarity, these two species are morphologically dissimilar [136]. *Neophaeothecoidea* is more closely related to *Haniomyces* in the phylogenetic results, but this relationship lacks statistical support. In addition, *Neophaeothecoidea* is reported as a hyphomycete [188], whereas *Haniomyces* produces a coelomycetous asexual morph.

Out of the 61 genera listed in Teratosphaeriaceae, only 24 genera are described with sexual morphs. We suggest that the sexual morphs of these genera require further examination with increased collections to verify the accurate treatment of and relationships to remaining species. During asexual stages of Teratosphaeriaceae, most members are pathogenic, whereas they are non-pathogenic during sexual stages. This is an important distinction for identifying opportunistic pathogens, as members of this family can easily spread diseases between locations. The new taxon, *Haniomyces dodonaeae*, fits morphologically well into Teratosphaeriaceae by its periphysate ostiole and hyaline ascospores with a single septum in each. However, the dimensions of the asci and ascospores are significantly larger than the existing sexual reports of this family. The golden brown, ellipsoidal conidia of *Haniomyces dodonaeae* are similar to those in *Neophaeothecoidea* and *Readeriella*. Phylogenetically, *Haniomyces dodonaeae* has a close proximity to *Neophaeothecoidea proteae*. This relationship, however, is not strongly supported in the ML and BI analyses (Figure 1). *Neophaeothecoidea proteae* was originally isolated as a coelomycete (*Phaeothecoidea proteae*) based on its yeast-like growth in culture [190]; however, it is currently accounted for in a hyphomycetous genus. Comparison of the 805 base pairs (bp) across the LSU gene region of *Haniomyces dodonaeae* shows 17 bp (2.1%) differences exist in comparison with *Neophaeothecoidea proteae*. Similarly, comparison of the 356 bp of the *rpb*2 gene region shows 56 bp (15.73%) differences in comparison with *Neophaeothecoidea proteae*.

*Rhytidhysteron* was introduced by Spegazzini [191] to account for *R*. *brasiliense* and *R*. *viride* collected from southern Brazil in 1877 and 1880, respectively. Spegazzini [56] did not designate any type; therefore, Clements and Shear [192] designated *R*. *brasiliense* as the type species. Subsequently, few species were introduced to this genus based on morphological evidence [193,194,195,196]. In recent studies, more species have been introduced based on both morphology and DNA-based sequence data [55,56,172,177,178,183]. Presently, there are 23 species mentioned in *Species Fungorum* [197], including saprobic to weakly pathogenic taxa that grow on woody plants in terrestrial habitats [56,181]. Species of *Rhytidhysteron* are typically involved in wood degradation and occur primarily on the woody parts of a broad range of hosts [64,188].

We introduce a new species into *Rhytidhysteron* from a dead twig of *Dodonaea* sp. in Honghe, China, and its relationships with other species are presented based on multigene phylogenetic analyses (Figure 2). Our analysed molecular data generated phylogenies consistent with those of Mapook et al. [55] and Hyde et al. [56]. The novel species, *Rhytidhysteron hongheense*, is phylogenetically closely related to *R*. *camporesii* (KUN-HKAS 104277) and *Rhytidhysteron chromolaenae* (MFLUCC 17-1516), and these three constitute a strongly supported monophyletic clade. The ascospore and asci characteristics between the three species are similar, but the colour of hysterothecia in *R*. *chromolaenae* (green) is different from the other two (black). The pseudo-epithecium of *R*. *camporesii* is brown to purple, whereas it is reddish orange in *R*. *hongheense*. The significance of these morphological characteristics in species delineation should be further investigated in terms of phylogenetic signals. A pairwise comparison of 521 ITS (+5.8S) sequence data showed 31 (5.95%) bp differences between *R*. *hongheense* and *R*. *camporesii* as well 28 (5.37%) bp differences between *R*. *hongheense* and *R*. *chromolaenae*. Currently, there are only two *Rhytidhysteron* species, viz. *Rhytidhysteron magnoliae* and *Rhytidhysteron thailandicum*, reported from China [56,198], making this report the third of its kind from China and first from Honghe Prefecture. 

Lophiostomataceae species are usually characterized by a slot-like ostiole on the top of the flattened neck, occurring mainly on twigs, stems or the bark of different woody and herbaceous plants in terrestrial, freshwater and marine environments as saprobes [129,140,188]. Thambugala et al. [129] undertook a comprehensive study of this family and accepted 16 genera. Subsequently, 12 new genera have been introduced by recent publications, and currently the family comprises 28 accepted genera [188]. The most recent multi-locus phylogenetic backbone tree to the family is presented in this study, including a novel genus (*Lophiomurispora*) found in Honghe County, Yunnan Province, China.

*Lophiomurispora* morphologically resembles *Coelodictyosporium*, *Platystomum* and *Sigarispora* with its crest-like ostiole and brown, multi-septate ascospores. However, these genera are revealed as phylogenetically distant in multi-gene phylogenetic analysis (Figure 3). *Lophiomurispora* has a close phylogenetic relationship to *Desertiserpentica* (Figure 3). However, *Desertiserpentica* is only known from its hyphomycetous asexual morph [54], whereas *Lophiomurispora* differs from *Desertiserpentica* by its coelomycetous asexual morph. Five strains of *Lophiomurispora* clustered in Lophiostomataceae as a strongly supported monophyletic clade (Figure 3) in both ML and BI of a concatenated SSU, LSU, ITS, *tef*1 and *rpb*2 dataset. All specimens were collected from dead twigs of *Dodonaea viscosa* at the Centre for Mountain Futures (CMF) in Honghe. There was no significant difference between morphological characteristics and DNA-based sequence comparisons between these collections. Therefore, we introduce them as different collections of *Lophiomurispora hongheensis.*

Parapyrenochaetaceae was proposed by Valenzuela-Lopez et al. [53] to accommodate three isolates which were previously recognized in *Pyrenochaeta*. They introduced the novel genus *Parapyrenochaeta* for *P*. *acaciae* (*Pyrenochaeta acaciae*), *P*. *protearum* (*Pyrenochaeta protearum*) and for the strain CBS 137997, formerly misidentified as *Pyrenochaeta pinicola* (re-identified as *Parapyrenochaeta protearum*). Later, Crous et al. [131] introduced *Quixadomyces* as another genus in Parapyrenochaetaceae to accommodate *Quixadomyces cearensis*. Therefore, there are currently two accepted genera in Parapyrenochaetaceae [3,188].

Crous et al. [131] introduced *Quixadomyces* for a fungus that was collected from Brazil on decaying bark. However, they did not observe the development of any internal structures. This fungus slightly resembles species in Pleosporales with its setose pycnidia [131,188]. In a multi-gene (concatenated LSU, SSU, ITS, *rpb*2, *tef*1 and *btub*) phylogenetic analysis, the ex-type strain of *Quixadomyces cearensis* (HUEFS 238438) clustered with two of our new strains as a monophyletic clade with poor bootstrap support (Figure 4). We introduce this isolate as a novel species belonging to this genus, *Q. hongheensis*. Based on the features of conidiogenous cells and conidia of *Quixadomyces hongheensis*, no substantial morphological differences exist to warrant two generic ranks. Therefore, this genus could potentially be reclassified as a synonym of *Parapyrenochaeta* in future studies. Because we did not perform extensive taxonomic reassessment using multiple fresh collections (especially sexual morphs of both genera), we will not attempt to synonymize any extant taxa.

Owing to lack of details on the internal structures of *Quixadomyces cearensis*, it is difficult to compare morphological characteristics such as conidiogenous cells and conidia between the new collection and this species. Lacking sufficient morphological evidence to perform accurate comparisons, we analysed nucleotide differences between these two strains. Comparing the 544 ITS (+5.8S) nucleotides of the two strains (HUEFS 238438 and KUMCC 20 0215) revealed 32 (5.88%) nucleotide differences. Therefore, it would seem prudent to treat our isolate as a new species in *Quixadomyces* as *Q*. *hongheensis*.

Nearly a century’s worth of taxonomic investigation into *Dodonaea viscosa* has yielded only 58 fungal records [Table 2]. These are mainly reported as saprobes or pathogens, but very few of these taxa are confirmed by both morphological and phylogenetic evidence. Many of these published records lack illustrations, descriptions or DNA sequence data, resulting in unclear taxonomic relationships. Even though *Dodonaea viscosa* is widely distributed across southwest and southern China, e.g., Fujian, Guangdong, Guangxi, Hainan, Sichuan and Yunnan [199], there is only one report for the fungus *Pseudocercospora mitteriana* on this host from China [124]. Previous taxonomic studies have suggested that increased collections might lead to the discovery of many new fungal species, and we, too, believe that *Dodonaea* is likely teeming with fungal diversity. More *Dodonaea* collections across different geographic regions are urgently needed, along with accompanying work in culture isolation, morphological description, DNA sequence analyses, phylogenetic relationship investigation, and accurate identification and classification. This study provides a case study for *Dodonaea viscosa* as a worthwhile host for the further study of microfungal associations and hints that it may potentially host numerous unknown fungal species.

## Figures and Tables

**Table 1:** Genes/loci used in the study with PCR primers, references and protocols.

Locus ^a^	Primers ^b^	PCR: Thermal Cycles: ^c^ (Annealing temp. in Bold)	References
*act*	ACT-512F ACT2Rd	(96 °C: 120 s, **52** °C: 60 s, 72 °C: 90 s) × 40 cycles	[41,42]
*btub*	TUB2Fw TUB4Rd	(94 °C: 30 s, **56** °C: 45 s, 72 °C: 60 s) × 35 cycles	[43]
*cal*	CAL-235F CAL2Rd	(96 °C: 120 s, **50** °C: 60 s, 72 °C: 90 s) × 40 cycles	[42,44]
ITS	ITS5 ITS4	(95 °C: 30 s, **55** °C:50 s, 72 °C: 90 s) × 35 cycles	[45]
LSU	LR0R LR5	(95 °C: 30 s, **55** °C:50 s, 72 °C: 90 s) × 35 cycles	[46,47]
*rpb*2	fRPB2-5f fRPB2-7cR	(94 °C: 60 s, **58** °C: 60 s, 72 °C: 90 s) × 40 cycles	[48]
	fRPB2-414R	(96 °C: 120 s, **49** °C: 60 s, 72 °C: 90 s) × 40 cycles	[49]
SSU	NS1 NS4	(95 °C: 30 s, **55** °C:50 s, 72 °C: 90 s) × 35 cycles	[45]
*tef*1	EF1-983F EF1-2218R	(95 °C: 30 s, **55** °C:50 s, 72 °C: 90 s) × 35 cycles	[50,51]
	EF1-728F EF-2	(96 °C: 120 s, **52** °C: 60 s, 72 °C: 90 s) × 40 cycles	[41,52]

^a^*act*: actin; *btub*: β-tubulin; *cal*: calmodulin; ITS: part of rDNA 18S (3’ end), the first internal transcribed spacer (ITS1), the 5.8S rRNA gene, the second ITS region (ITS2), and part of the 28S rRNA (5’ end); LSU: large subunit (28S); *rpb*2: RNA polymerase II second largest subunit; SSU: small subunit rDNA (18S); *tef*1: translation elongation factor 1-alpha gene. ^b^ fRPB2-5f and fRPB2-414R were used only for Teratosphaeriaceae analysis. ^c^ All the PCR thermal cycles include initiation step of 95 °C: 5 min, and final elongation step of 72 °C: 10 min and final hold at 4 °C.

**Table 2:** Checklist of fungi recorded from *Dodonaea viscosa* in worldwide.

Phylum and Class	Order	Family	Species	Country	References
**Ascomycota**					
Dothideomycetes	Botryosphaeriales	Botryosphaeriaceae	*Lasiodiplodia iraniensis*	Australia	[65]
			*Macrophoma dodonaeae*	India	[66]
			*Macrophomina phaseolina*	Arizona	[67]
	Capnodiales	Capnodiaceae	*Antennariella californica*	Fiji	[68]
		Mycosphaerellaceae	*Cercospora dodonaeae*	India	[69,70,71]
			*Cercospora* sp.	Sierra Leone	[72]
			*Pseudocercospora dodonaeae*	New Zealand	[73,74,75,76,77,78]
			*Pseudocercospora mitteriana*	China	[79]
				India	[69,71]
				Pakistan	[71,80]
		Teratosphaeriaceae	*Haniomyces dodonaeae*	China	This study
	Hysteriales	Hysteriaceae	*Rhytidhysteron hongheense*	China	This study
	incertae sedis	Pseudoperisporiaceae	*Episphaerella dodonaeae*	Dominican Republic	[81]
				Ecuador	[82]
				Venezuela	[82]
				USA	[83]
		incertae sedis	*Mycothyridium pakistanicum*	Pakistan	[80]
			*Mycothyridium roosselianum*	Pakistan	[80]
	Patellariales	Patellariaceae	*Tryblidaria pakistani*	Pakistan	[80]
	Pleosporales	Coniothyriaceae	*Coniothyrium* sp.	Venezuela	[84]
		Corynesporascaceae	*Corynespora cassiicola*	India	[85]
		Didymosphaeriaceae	*Didymosphaeria oblitescens*	Pakistan	[80]
		Leptosphaeriaceae	*Leptosphaeria dodonaeae*	Eritrea	[86]
		Lophiostomataceae	*Lophiomurispora hongheensis*	China	This study
		Parapyrenochaetaceae	*Quixadomyces hongheensis*	China	This study
		Pleosporaceae	*Pleospora dodonaeae*	Cyprus	[87]
	Valsariales	Valsariaceae	*Valsaria rubricosa*	Pakistan	[80]
Lecanoromycetes	Ostropales	Stictidaceae	*Stictis marathwadensis*	India	[88,89]
Leotiomycetes	Helotiales	Erysiphaceae	*Oidium* sp.	Iraq	[90]
				Israel	[90]
				South Africa	[90]
				Zimbabwe	[91]
			*Ovulariopsis erysiphoides*	Ethiopia	[92]
			*Phyllactinia*sp.	Ethiopia	[90]
			*Sawadaea bicornis*	Germany	[93]
				New Zealand	[74,90]
				South Africa	[90]
			*Takamatsuella circinata*	South Africa	[94]
Sordariomycetes	Diaporthales	Cytosporaceae	*Cytospora* sp.	USA	[95]
	Glomerellales	Glomerellaceae	*Colletotrichum gloeosporioides*	India	[88]
	Meliolales	Meliolaceae	*Meliola lyoni*	Hawaii	[96,97,98,99]
	Hypocreales	Nectriaceae	*Calonectria cylindrospora*	USA	[100,101]
			*Calonectria pauciramosa*	Italy	[102]
			*Fusarium solani*	Iran	[103]
	Glomerellales	Plectosphaerellaceae	*Verticillium dahliae*	USA	[95]
				New Zealand	[74]
	Coronophorales	Scortechiniaceae	*Tympanopsis lantanae*	India	[104]
	Amphisphaeriales	Sporocadaceae	*Monochaetia dodoneae*	Ethiopia	[92]
			*Pestalotia dodonaeae*	Eritrea	[86]
			*Sarcostroma kennedyae*	New Zealand	[74]
			*Seimatosporium kennedyae*	New Zealand	[73]
	Togniniales	Togniniaceae	*Phaeoacremonium alvesii*	Australia	[105,106,107,108]
			*Phaeoacremonium italicum*	Australia	[109]
**Basidiomycota**					
Agaricomycetes	Agaricales	Marasmiaceae	*Campanella junghuhnii*	Hawaii	[110]
	Agaricales	incertae sedis	*Dendrothele incrustans*	New Zealand	[111]
Bartheletiomycetes	Cantharellales	Ceratobasidiaceae	*Rhizoctonia* sp.	Italy	[112]
	Hymenochaetales	Hymenochaetaceae	*Arambarria cognata*	Uruguay	[113]
			*Fomitiporia australiensis*	Australia	[114]
			*Phellinus melleoporus*	Hawaii	[110]
			*Phellinus robustus*	USA	[115]
			*Phellinus sonorae*	USA	[116]
		Schizoporaceae	*Hyphodontia alutaria*	Hawaii	[110]
			*Grandinia breviseta*	Hawaii	[110]
	Polyporales	Hyphodermataceae	*Hyphoderma sphaeropedunculatum*	Hawaii	[110]
Pucciniomycetes	Pucciniales	incertae sedis	*Uredo dodonaeae*	Indonesia	[117]
**Oomycota**					
Peronosporomycetes	Peronosporales	Peronosporaceae	*Phytophthora drechsleri*	Australia	[118,119,120]
			*Phytophthora nicotianae*	Italy	[121,122,123]
			*Phytophthora palmivora*	Italy	[123]
		Pythiaceae	*Globisporangium debaryanum*	New Zealand	[73,74]
			*Globisporangium irregulare*	New Zealand	[74]
			*Globisporangium ultimum*	New Zealand	[73]
			*Pythium inflatum*	New Zealand	[73,74]
			*Pythium* sp.	New Zealand	[73]
				USA	[75]

**Table 3:** Taxa used in the phylogenetic analyses and their corresponding GenBank numbers.

Species	Strain	GenBank Accession Numbers								Reference
		SSU	LSU	*act*	*cal*	ITS	*rpb*2	*tef*1	*btub*	
*Acidiella bohemica*	CBS 132720	-	KF901984	-	-	-	KF902178	-	-	[49]
*Acidiella parva*	CMW 10189	-	KF901986	KF903512	KF902537	KF901647	KF902192	KF903097 *	-	[49]
*Acrodontium crateriforme*	CPC 11509	-	GU214682	GU320413	KX289011	GU214682	KX288404	GU384425 *	-	[124,125]
*Acrodontium pigmentosum*	CBS 111111	-	KX286963	-	-	KX287275	KX288412	-	-	[125]
*Alfoldia vorosii*	CBS 145501	MK589346	MK589354	-	-	JN859336	-	MK599320	-	[126]
*Alpestrisphaeria jonesii*	GZCC 16-0021	KX687755	KX687753	-	-	KX687757	-	KX687759	-	[14]
*Alpestrisphaeria jonesii*	GZCC 16-0022	KX687756	KX687754	-	-	KX687758	-	KX687760	-	[14]
*Alpestrisphaeria monodictyoides*	V0216		MH160808	-	-	MK503662	-		-	[127]
*Alpestrisphaeria terricola*	SC-12H	JX985749	JX985750	-	-	JN662930	-		-	[128]
*Amorocoelophoma cassiae*	MFLUCC 17-2283	NG_065775	NG_066307	-	-	NR_163330	MK434894	MK360041	-	[127]
*Angustimassarina acerina*	MFLUCC 14-0505	NG_063573	KP888637	-	-	NR_138406	-	KR075168	-	[129]
*Angustimassarina quercicola*	MFLUCC 14-0506	NG_063574	KP888638	-	-	KP899133	-	KR075169	-	[129]
*Angustimassarina rosarum*	MFLUCC 17-2155	MT226662	MT214543	-	-	MT310590	MT394678	MT394726	-	[130]
*Apenidiella strumelloidea*	CBS 114484	-	KF937229	-	-	-	KF937266	-	-	[49]
*Araucasphaeria foliorum*	CPC 33084	-	MH327829	-	-	MH327793	-	-	-	[131]
*Astragalicola vasilyevae*	MFLUCC 17-0832	MG829098	MG828986	-	-	NR_157504	MG829248	MG829193	-	[130]
*Austroafricana associata*	CPC 13119	-	KF901824	KF903526	KF902528	KF901507	KF902177	KF903087 *	-	[49]
*Austroafricana* sp.	CPC 4313	-	KF901813	KF903460	KF902527	KF901498	KF902186	KF903086 *	-	[49]
*Austrostigmidium mastodiae*	MA 18215	-	NG_057063	-	-	-	-	-	-	[132]
*Austrostigmidium mastodiae*	MA 18213	-	KP282862	-	-	-	-	-	-	[132]
*Batcheloromyces alistairii*	CPC 12730	-	KF937220	-	-	-	KF937252	-	-	[49]
*Batcheloromyces leucadendri*	CPC 1838	-	KF937221	-	-	-	KF937253	-	-	[49]
*Batcheloromyces sedgefieldii*	CPC 3026	-	KF937222	-	-	-	KF937254	-	-	[49]
*Biappendiculispora japonica*	KT 573	AB618686	AB619005	-	-	LC001728	-	LC001744	-	[129,133]
*Biappendiculispora japonica*	KT 686-1	AB618687	AB619006	-	-	LC001729	-	LC001745	-	[129,133]
*Camarosporidiella caraganicola*	MFLUCC 17-0726	MF434300	MF434212	-	-	MF434125	-	MF434388	-	[134]
*Camarosporidiella elongata*	AFTOL-ID 1568	DQ678009	DQ678061	-	-	-	DQ677957	DQ677904	-	[135]
*Camarosporidiella eufemiana*	MFLUCC 17-0207	MF434321	MF434233	-	-	MF434145	-	MF434408	-	[134]
*Camarosporula persooniae*	CPC 3350	-	JF770460	-	-	-	KF937255	-	-	[49,136]
*Capulatispora sagittiformis*	KT 1934	AB618693	AB369267	-	-	AB369268	-	LC001756	-	[129,133]
*Catenulostroma hermanusense*	CPC 18276	-	KF902089	-	-	-	KF902197	-	-	[49]
*Catenulostroma protearum*	CPC 15370	-	KF902090	-	-	-	KF902198	-	-	[49]
*Coelodictyosporium pseudodictyosporium*	MFLUCC 13-0451	-	KR025862	-	-	KR025858	-	-	-	[137]
*Coelodictyosporium rosarum*	MFLUCC 17-0776	NG_063674	NG_059056	-	-	MG828875	-	MG829195	-	[130]
*Coniothyrium palmarum*	CBS 400.71	EU754054	JX681084	-	-	MH860184	KT389592	-	KT389792	[138]
*Constantinomyces macerans*	TRN 440	-	KF310005	-	-	NR_164011	KF310081	-	-	[139]
*Constantinomyces minimus*	CBS 118766	-	KF310003	-	-	NR_144957	KF310077	-	-	[139]
*Crassiclypeus aquaticus*	KH 91	LC312469	LC312527	-	-	LC312498	LC312585	LC312556	-	[140]
*Crassiclypeus aquaticus*	KH 104	LC312470	LC312528	-	-	LC312499	LC312586	LC312557	-	[140]
*Crassiclypeus aquaticus*	KH 185	LC312471	LC312529	-	-	LC312500	LC312587	LC312558	-	[140]
*Crassiclypeus aquaticus*	KT 970	LC312472	LC312530	-	-	LC312501	LC312588	LC312559	-	[140]
*Desertiserpentica hydei*	SQUCC 15092	MW077163	MW077156	-	-	MW077147	MW075773	MW077163	-	[54]
*Devriesia agapanthi*	CPC 19833	-	JX069859	-	-	-	KJ564346	-	-	[49,141]
*Devriesia strelitziae*	X1037	-	GU301810	-	-	EU436763	GU371738	GU349049 *	-	[142]
*Dimorphiopsis brachystegiae*	CPC 22679	-	KF777213	-	-	KF777160	-	-	-	[143]
*Elasticomyces elasticus*	CCFEE 5313	-	KJ380894	-	-	FJ415474	-	-	-	[49,144]
*Elasticomyces elasticus*	CCFEE 5474	-	KF309991	-	-	-	KF310046	-	-	[139]
*Eupenidiella venezuelensis*	CBS 106.75	-	KF902163	KF903393	KF902540	KF901802	KF902202	KF903100 *	-	[49]
*Euteratosphaeria verrucosiafricana*	CPC 11167	-	-	-	-	DQ303056	-	-	-	[139]
*Flabellascoma aquaticum*	KUMCC 15-0258	MN304832	NG_068307	-	-	NR_166305	MN328895	MN328898	-	[145]
*Flabellascoma cycadicola*	KT 2034	LC312473	LC312531	-	-	LC312502	LC312589	LC312560	-	[140]
*Flabellascoma fusiforme*	MFLUCC 18-1584	-	NG_068308	-	-	NR_166306	-	MN328902	-	[105]
*Flabellascoma minimum*	KT 2013	LC312474	LC312532	-	-	LC312503	LC312590	LC312561	-	[140]
*Flabellascoma minimum*	KT 2040	LC312475	LC312533	-	-	LC312504	LC312591	LC312562	-	[140]
*Forliomyces uniseptata*	MFLUCC 15-0765	NG_061234	NG_059659	-	-	NR_154006	-	KU727897	-	[146]
*Friedmanniomyces endolithicus*	CCFEE 5199	-	KF310007	-	-	-	KF310093	-	-	[139]
*Friedmanniomyces endolithicus*	CCFEE 5283	-	KF310006	-	-	-	KF310053	-	-	[49]
*Gloniopsis calami*	MFLUCC 15-0739	NG_063621	NG_059715	-	-	NR_164398	-	KX671965	-	[147]
*Gloniopsis calami*	MFLUCC 10-0927	MN577426	MN577415	-	-	MN608546	-	-	-	[148]
*Gloniopsis praelonga*	CBS 112415	FJ161134	FJ161173	-	-	-	FJ161113	FJ161090	-	[149]
*Guttulispora crataegi*	MFLUCC 13-0442	KP899125	KP888639	-	-	KP899134	-	KR075161	-	[129]
*Guttulispora crataegi*	MFLUCC 14-0993	KP899126	KP888640	-	-	KP899135	-	KR075162	-	[129]
***Haniomyces dodonaeae***	**KUMCC 20-0220**	**MW264221**	**MW264191**	**MW256802**	**MW256805**	**MW264212**	**MW269527**	MW256813 *	**-**	**This study**
***Haniomyces dodonaeae***	**KUMCC 20-0221**	**MW264222**	**MW264192**	**MW256803**	**MW256806**	**MW264213**	**MW269528**	MW256814 *	**-**	**This study**
*Hortaea thailandica*	CPC 16651	-	KF902125	-	-	-	KF902206	-	-	[49]
*Hysterium angustatum*	MFLUCC 16-0623	MH535885	MH535893	-	-	-	MH535875	FJ161096	-	[149,150]
*Hyweljonesia indica*	NFCCI 4146	-	NG_066398	-	-	NR_164021	-	-	-	[151]
*Hyweljonesia queenslandica*	BRIP 61322b	-	NG_059766	-	-	NR_154095	-	-	-	[152]
*Incertomyces perditus*	CCFEE 5385	-	KF310008	-	-	KF309977	KF310083	-	-	[139]
*Incertomyces vagans*	CCFEE 5393	-	KF310009	-	-	NR_154064	KF310057	-	-	[139]
*Lapidomyces hispanicus*	TRN126	-	KF310016	-	-	-	KF310076	-	-	[139]
*Lentistoma bipolare*	HKUCC 10069	LC312476	LC312534	-	-	LC312505	LC312592	LC312563	-	[140]
*Lentistoma bipolare*	HKUCC 10110	LC312477	LC312535	-	-	LC312506	LC312593	LC312564	-	[140]
*Lentistoma bipolare*	HKUCC 8277	LC312478	LC312536	-	-	LC312507	LC312594	LC312565	-	[140]
*Lentistoma bipolare*	KT 2415	LC312483	LC312541	-	-	LC312512	LC312599	LC312570	-	[140]
*Lentistoma bipolare*	KT 3056	LC312484	LC312542	-	-	LC312513	LC312600	LC312571	-	[140]
*Leptoparies palmarum*	KT 1653	LC312485	LC312543	-	-	LC312514	LC312601	LC312572	-	[140]
*Leptosphaeria conoidea*	CBS 616.75	JF740099	JF740279	-	-	JF740201	KT389639	-	KT389804	[153]
*Leptosphaeria doliolum*	CBS 505.75	NG_062778	NG_068574	-	-	NR_155309	KY064035	GU349069	JF740144	[154]
*Lophiohelichrysum helichrysi*	MFLUCC 15-0701	KT333437	KT333436	-	-	KT333435	-	KT427535	-	[155]
*Lophiopoacea paramacrostoma*	MFLUCC 11-0463	KP899122	KP888636	-	-	-	-	-	-	[129]
***Lophiomurispora hongheensis***	**KUMCC 20-0217**	**MW264225**	**MW264195**	**-**	**-**	**MW264216**	**MW256808**	**MW256817**	**-**	**This study**
***Lophiomurispora hongheensis***	**KUMCC 20-0223**	**MW264226**	**MW264196**	**-**	**-**	**MW264217**	**MW256809**	**MW256818**	**-**	**This study**
***Lophiomurispora hongheensis***	**KUMCC 20-0216**	**MW264227**	**MW264197**	**-**	**-**	**MW264218**	**MW256810**	**MW256819**	**-**	**This study**
***Lophiomurispora hongheensis***	**KUMCC 20-0219**	**MW264228**	**MW264198**	**-**	**-**	**MW264219**	**MW256811**	**MW256820**	**-**	**This study**
***Lophiomurispora hongheensis***	**KUMCC 20-0224**	**MW264229**	**MW264199**	**-**	**-**	**MW264220**	**MW256812**	**MW256821**	**-**	**This study**
*Lophiopoacea winteri*	KT 740	AB618699	AB619017	-	-	JN942969	JN993487	LC001763	-	[129,133,156]
*Lophiopoacea winteri*	KT 764	AB618700	AB619018	-	-	JN942968	JN993488	LC001764	-	[129,133,156]
*Lophiostoma caulium*	CBS 623.86	GU296163	GU301833	-	-	-	GU371791	-	-	[152]
*Lophiostoma macrostomum*	KT 635	AB521731	AB433273	-	-	AB433275	JN993484	LC001752	-	[129,133]
*Lophiostoma multiseptatum*	JCM 17668	AB618684	AB619003	-	-	LC001726	-	LC001742	-	[129,133]
*Lophiostoma multiseptatum*	MAFF 239451	AB618685	AB619004	-	-	LC001727	-	LC001743	-	[129,133]
*Lophiostoma rosae*	TASM 6115	NG_065145	NG_069558	-	-	NR_158531	-	MG829205	-	[130]
*Lophiostoma semiliberum*	KT 828	AB618696	AB619014	-	-	JN942970	JN993489	LC001759	-	[129,133,156]
*Massarina cisti*	CBS 266.62	AB797249	AB807539	-	-	LC014568	FJ795464	AB808514	-	[157,158]
*Massarina eburnea*	CBS 473.64	GU296170	GU301840	-	-	AF383959	GU371732	GU349040	-	[143,159]
*Meristemomyces frigidum*	CCFEE 5457	-	GU250389	-	-	-	KF310066	-	-	[49,144]
*Meristemomyces frigidum*	CCFEE 5507	-	KF310013	-	-	-	KF310067	-	-	[139]
*Monticola elongata*	CCFEE 5492	-	KF309994	-	-	-	KF310065	-	-	[139]
*Myrtapenidiella corymbia*	CPC 14640	-	KF901838	KF903558	KF902558	KF901517	KF902227	KF903119 *	-	[49]
*Neocatenulostroma abietis*	CBS 110038	-	KF937226	-	-	-	KF937263	-	-	[49]
*Neocatenulostroma microsporum*	CPC 1960	-	KF901814	-	KF902561	KF901499	KF902232	KF903122 *	-	[49]
*Neocucurbitaria ribicola*	CBS 142394	MF795840	MF795785	-	-	MF795785	MF795827	MF795873	MF795911	[160]
*Neoleptosphaeria jonesii*	MFLUCC 16-1442	NG_063625	KY211870	-	-	NR_152375	-	KY211872	-	[161]
*Neopaucispora rosaecae*	MFLUCC 17-0807	NG_061293	NG_059869	-	-	MG828924	-	MG829217	-	[130]
*Neophaeosphaeria agaves*	CBS 136429	-	KF777227	-	-	NR_137833	-	-	-	[143]
*Neophaeosphaeria filamentosa*	CBS 102202	GQ387516	GQ387577	-	-	JF740259	GU371773	-	-	[162]
*Neophaeosphaeria phragmiticola*	KUMCC 16-0216	MG837008	MG837009	-	-	-	-	MG838020	-	[163]
*Neophaeothecoidea proteae*	CPC 2831	-	KF937228	-	-	-	KF937265	-	-	[49]
*Neopyrenochaeta acicola*	CBS 812.95	NG_065567	GQ387602	-	-	NR_160055	LT623271	-	LT623232	[164]
*Neopyrenochaeta cercidis*	MFLU 18-2089	NG_065769	MK347932	-	-	MK347718	MK434908	-	-	[127]
*Neopyrenochaeta fragariae*	CBS 101634	GQ387542	GQ387603	-	-	LT623217	LT623270	-	LT623231	[164]
*Neopyrenochaeta inflorescentiae*	CBS 119222	-	EU552153	-	-	EU552153	LT623272	-	LT623233	[165]
*Neopyrenochaeta maesuayensis*	MFLUCC 14-0043	-	MT183504	-	-	NR_170043	-	MT454042	-	[166]
*Neopyrenochaeta telephoni*	CBS 139022	-	NG_067485	-	-	KM516291	LT717685	-	LT717678	[154]
*Neotrematosphaeria biappendiculata*	KT 1124	GU205256	GU205227	-	-	-	-	-	-	[129]
*Neotrematosphaeria biappendiculata*	KT 975	GU205254	GU205228	-	-	-	-	-	-	[129]
*Neotrimmatostroma excentricum*	CPC 13092	-	KF901840	KF903534	KF902562	KF901518	KF902236	KF903123 *	-	[49]
*Neovaginatispora clematidis*	MFLUCC 17-2149	MT226676	MT214559	-	-	MT310606	-	MT394738	-	[167]
*Neovaginatispora fuckelii*	CBS 101952	FJ795496	DQ399531	-	-	-	FJ795472	-	-	[158]
*Neovaginatispora fuckelii*	KH 161	AB618689	AB619008	-	-	LC001731	-	LC001749	-	[129,133]
*Neovaginatispora fuckelii*	KT 634	AB618690	AB619009	-	-	LC001732	-	LC001750	-	[129,133]
*Oleoguttula mirabilis*	CCFEE 5522	-	KF310019	-	-	-	KF310070	-	-	[139]
*Parapaucispora pseudoarmatispora*	KT 2237	LC100018	LC100026	-	-	LC100021	-	LC100030	-	[168]
*Parapenidiella pseudo tasmaniensis*	CPC 12400	-	KF901844	KF903562	KF902589	KF901522	KF902265	KF903152 *	-	[49]
*Parapenidiella tasmaniensis*	CPC 1555	-	KF901843	KF903451	KF902587	KF901521	KF902263	KF903150 *	-	[49]
*Parapyrenochaeta acaciae*	CPC 25527	-	KX228316	-	-	NR_155674	LT717686	-	LT717679	[53]
*Parapyrenochaeta protearum*	CBS 131315	-	JQ044453	-	-	JQ044434	LT717683	-	LT717677	[53]
*Paucispora kunmingense*	MFLUCC 17-0932	MF173430	NG_059829	-	-	NR_156625	MF173436	MF173434	-	[169]
*Paucispora quadrispora*	KH 448	LC001720	LC001722	-	-	LC001733	-	LC001754	-	[129]
*Paucispora quadrispora*	KT 843	AB618692	AB619011	-	-	LC001734	-	LC001755	-	[129,133]
*Paucispora versicolor*	KH 110	LC001721	AB918732	-	-	AB918731	-	LC001760	-	[129,133]
*Penidiella columbiana*	CBS 486.80	-	KF901965	KF903587	KF902594	KF901630	KF902272	KF903158 *	-	[49]
*Penidiellomyces aggregatus*	CBS 128772	-	NG_057905	-	-	NR_137772	-	-	-	[170]
*Penidiellomyces drakensbergensis*	CPC 19778	-	NG_059482	-	-	NR_111821	-	-	-	[141]
*Penidiellopsis radicularis*	CBS 131976	-	KU216314	-	KU216292	KT833148	-	KU216339 *	-	[171]
*Penidiellopsis ramosus*	CBMAI 1937	-	KU216317	-	KU216295	KT833151	-	KU216342 *	-	[171]
*Phaeoseptum* *carolshearerianum*	NFCCI-4221	MK307816	MK307813	-	-	MK307810	MK309877	MK309874	-	[172]
*Phaeoseptum hydei*	MFLUCC 17-0801	MT240624	MT240623	-	-	MT240622	-	MT241506	-	[40]
*Phaeoseptum manglicola*	NFCCI-4666	MK307817	MK307814	-	-	MK307811	MK309878	MK309875	-	[172]
*Phaeoseptum terricola*	MFLUCC 10-0102	MH105780	MH105779	-	-	MH105778	MH105782	MH105781	-	[163]
*Phaeothecoidea Intermedia*	CPC 13711	-	KF902106	KF903564	KF902606	KF901752	KF902286	KF903171 *	-	[49]
*Phaeothecoidea Minutispora*	CPC 13710	-	KF902108	KF903659	KF902607	KF901753	KF902288	KF903172 *	-	[49]
*Piedraia hortae var. hortae*	CBS 480.64	-	KF901943	-	-	-	KF902289	-	-	[49]
*Piedraia hortae var. paraguayensis*	CBS 276.32	-	KF901816	-	-	-	-	-	-	[49]
*Piedraia quintanilhae*	CBS 327.63	-	KF901957	-	-	-	-	-	-	[49]
*Platystomum actinidiae*	KT 521	JN941375	JN941380	-	-	JN942963	JN993490	LC001747	-	[129,156]
*Platystomum crataegi*	MFLUCC 14-0925	KT026113	KT026109	-	-	KT026117	-	KT026121	-	[129]
*Platystomum rosae*	MFLU 15-2569	KY264750	KY264746	-	-	KY264742	-	-	-	[173]
*Platystomum rosae*	MFLUCC 15-0633	KT026115	KT026111	-	-	KT026119	-	-	-	[129]
*Platystomum salicicola*	MFLUCC 15-0632	KT026114	KT026110	-	-	KT026118	-	-	-	[129]
*Pseudolophiostoma cornisporum*	KH 322	LC312486	LC312544	-	-	LC312515	LC312602	LC312573	-	[140]
*Pseudolophiostoma obtusisporum*	KT 2838	LC312489	LC312547	-	-	LC312518	LC312605	LC312576	-	[140]
*Pseudolophiostoma obtusisporum*	KT 3119	LC312491	LC312549	-	-	LC312520	LC312607	LC312578	-	[140]
*Pseudolophiostoma tropicum*	KH 352	LC312492	LC312550	-	-	LC312521	LC312608	LC312579	-	[140]
*Pseudolophiostoma tropicum*	KT 3134	LC312493	LC312551	-	-	LC312522	LC312609	LC312580	-	[140]
*Pseudopaucispora brunneospora*	KH 227	LC312494	LC312552	-	-	LC312523	LC312610	LC312581	-	[140]
*Pseudoplatystomum scabridisporum*	BCC 22835	GQ925831	GQ925844	-	-	-	GU479830	GU479857	-	[174]
*Pseudoplatystomum scabridisporum*	BCC 22836	GQ925832	GQ925845	-	-	-	GU479829	GU479856	-	[174]
*Pseudopyrenochaeta lycopersici*	CBS 306.65	NG_062728	MH870217	-	-	NR_103581	LT717680	-	LT717674	[154]
*Pseudopyrenochaeta terrestris*	CBS 282.72	-	LT623216	-	-	LT623228	LT623287	-	LT623246	[53]
*Pseudoteratosphaeria flexuosa*	CPC 673	-	KF902098	KF903403	KF902653	KF901745	KF902345	KF903228 *	-	[49]
*Pseudoteratosphaeria flexuosa*	CPC 1109	-	KF902110	KF903421	KF902654	KF901755	KF902346	-	-	[49]
*Pyrenochaeta nobilis*	CBS 407.76	DQ898287	EU754206	-	-	NR_103598	DQ677991	DQ677936	MF795916	[162]
*Pyrenochaeta pinicola*	CBS 137997	-	KJ869209	-	-	KJ869152	LT717684	-	KJ869249	[175]
*Pyrenochaeta sp.*	DTO 305-C6	-	KX171361	-	-	KX147606	-	-	-	[176]
*Pyrenochaetopsis botulispora*	CBS 142458	-	LN907440	-	-	LT592945	LT593084	-	LT593014	[53]
*Pyrenochaetopsis globosa*	CBS 143034	-	LN907418	-	-	LT592934	LT593072	-	LT593003	[53]
*Pyrenochaetopsis paucisetosa*	CBS 142460	-	LN907336	-	-	LT592897	LT593035	-	LT592966	[53]
*Pyrenochaetopsis setosissima*	CBS 119739	-	GQ387632	-	-	LT623227	LT623285	-	LT623245	[162]
*Queenslandipenidiella kurandae*	CPC 13333	-	KF901860	KF903538	KF902663	KF901538	KF902356	KF903238 *	-	[49]
*Quixadomyces cearensis*	HUEFS 238438	-	NG_066409	-	-	NR_160606	-	-	-	[131]
***Quixadomyces hongheensis***	**KUMCC 20-0215**	**MW264223**	**MW264193**	**-**	**-**	**MW264214**	**MW269529**	**MW256815**	**MW256804**	**This study**
***Quixadomyces hongheensis***	**HKAS112346**	**MW541833**	**MW541822**	**-**	**-**	**MW541826**	**MW556136**	**MW556134-**	**MW556137**	**This study**
***Quixadomyces hongheensis***	**HKAS112347**	**MW541834**	**MW541823**	**-**	**-**	**MW541827**	**-**	**MW556135-**	**MW556138**	**This study**
*Ramusculicola clematidis*	MFLUCC 17-2146	NG_070667	MT214596	-	-	MT310640	MT394707	MT394652	-	[167]
*Readeriella angustia*	CPC 13608	-	KF902114	KF903566	KF902669	KF901759	KF902364	KF903246 *	-	[49]
*Readeriella deanei*	CPC 12715	-	KF901864	KF903583	KF902673	KF901542	KF902368	KF903250 *	-	[49]
*Readeriella dimorphospora*	CPC 12636	-	KF901866	KF903622	KF902675	KF901544	KF902370	KF903252 *	-	[49]
*Readeriella menaiensis*	CPC 14447	-	KF901870	KF903572	KF902678	KF901548	KF902374	KF903256 *	-	[49]
*Recurvomyces mirabilis*	CCFEE 5264	-	GU250372	-	-	-	KF310059	-	-	[139,144]
*Recurvomyces mirabilis*	CCFEE 5475	-	KC315876	-	-	-	KF310060	-	-	[139,144]
*Rhytidhysteron bruguierae*	MFLUCC 17-1502	MN632464	MN632453	-	-	MN632458	-	MN635662	-	[55]
*Rhytidhysteron bruguierae*	MFLUCC 17-1515	MN632463	MN632452	-	-	MN632457	-	MN635661	-	[55]
*Rhytidhysteron bruguierae*	MFLUCC 18-0398	MN017901	MN017833	-	-	-	-	MN077056	-	[172]
*Rhytidhysteron bruguierae*	MFLUCC 17-1511	MN632465	MN632454	-	-	MN632459	-	-	-	[55]
*Rhytidhysteron camporesii*	HKAS 104277		MN429072	-	-	MN429069	-	MN442087	-	[148]
*Rhytidhysteron chromolaenae*	MFLUCC 17-1516	NG_070139	NG_068675	-	-	MN632461	-	MN635663	-	[55]
*Rhytidhysteron erioi*	MFLU 16-0584	-	MN429071	-	-	MN429068	-	MN442086	-	[148]
***Rhytidhysteron hongheense***	**KUMCC 20-0222**	**MW264224**	**MW264194**	**-**	**-**	**MW264215**	**MW256807**	**MW256816**	**-**	**This study**
***Rhytidhysteron hongheense***	**HKAS112348**	**MW541831**	**MW541820**	**-**	**-**	**MW541824**	**-**	**MW556132**	**-**	**This study**
***Rhytidhysteron hongheense***	**HKAS112349**	**MW541832**	**MW541821**	**-**	**-**	**MW541825**	**-**	**MW556133**	**-**	**This study**
*Rhytidhysteron hysterinum*	EB 0351	-	GU397350	-	-	-	-	GU397340	-	[149]
*Rhytidhysteron hysterinum*	CBS 316.71	-	MH871912	-	-	MH860141	-	-	-	[154]
*Rhytidhysteron magnoliae*	MFLUCC 18-0719	MN989382	MN989384	-	-	MN989383	-	MN997309	-	[177]
*Rhytidhysteron mangrovei*	MFLUCC 18-1113	-	NG_067868	-	-	NR_165548	-	MK450030	-	[178]
*Rhytidhysteron neorufulum*	MFLUCC 13-0216	KU377571	KU377566	-	-	KU377561	-	KU510400	-	[177]
*Rhytidhysteron neorufulum*	GKM 361A	GU296192	GQ221893	-	-	-	-	-	-	[179]
*Rhytidhysteron neorufulum*	HUEFS 192194	-	KF914915	-	-	-	-	-	-	[180]
*Rhytidhysteron neorufulum*	MFLUCC 12-0528	KJ418119	KJ418117	-	-	KJ418118	-	-	-	[181]
*Rhytidhysteron neorufulum*	CBS 306.38	AF164375	FJ469672	-	-	-	-	GU349031	-	[142]
*Rhytidhysteron neorufulum*	MFLUCC 12-0011	KJ418110	KJ418109	-	-	KJ206287	-	-	-	[181]
*Rhytidhysteron neorufulum*	MFLUCC 12-0567	KJ546129	KJ526126	-	-	KJ546124	-	-	-	[181]
*Rhytidhysteron neorufulum*	MFLUCC 12-0569	KJ546131	KJ526128	-	-	KJ546126	-	-	-	[181]
*Rhytidhysteron neorufulum*	MFLUCC 14-0577	KU377570	KU377565	-	-	KU377560	-	KU510399	-	[177]
*Rhytidhysteron opuntiae*	GKM 1190		GQ221892	-	-	-	-	GU397341	-	[179]
*Rhytidhysteron rufulum*	EB 0384	GU397368	GU397354	-	-	-	-	-	-	[182]
*Rhytidhysteron rufulum*	EB 0382	GU397367	GU397352	-	-	-	-	-	-	[182]
*Rhytidhysteron rufulum*	EB 0383		GU397353	-	-	-	-	-	-	[182]
*Rhytidhysteron rufulum*	MFLUCC 12-0013	KJ418113	KJ418111	-	-	KJ418112	-	-	-	[181]
*Rhytidhysteron tectonae*	MFLUCC 13-0710	KU712457	KU764698	-	-	KU144936	-	KU872760	-	[183]
*Rhytidhysteron thailandicum*	MFLUCC 13-0051		MN509434	-	-	MN509433	-	MN509435	-	[56]
*Rhytidhysteron thailandicum*	MFLUCC 12-0530	KJ546128	KJ526125	-	-	KJ546123	-	-	-	[172]
*Rhytidhysteron thailandicum*	MFLUCC 14-0503	KU377569	KU377564	-	-	KU377559	-	KU497490	-	[177]
*Seltsamia ulmi*	CBS 143002	MF795794	MF795794	-	-	MF795794	MF795836	MF795882	MF795918	[160]
*Sigarispora arundinis*	KT 651	AB618680	AB618999	-	-	JN942965	JN993486	LC001738	-	[129,133]
*Sigarispora caudata*	MAFF 239453	AB618681	AB619000	-	-	LC001723	-	LC001739	-	[129,133]
*Sigarispora caulium*	MAFF 239450	AB618682	AB619001	-	-	LC001724	-	LC001740	-	[129,133]
*Sigarispora caulium*	JCM 17669	AB618683	AB619002	-	-	LC001725	-	LC001741	-	[129,133]
*Sigarispora ononidis*	MFLUCC 15-2667	KU243126	KU243125	-	-	KU243128	-	KU243127	-	[169]
*Sigarispora rosicola*	MFLU 15-1888	NG_062116	MG829080	-	-	MG828968	-	MG829240	-	[130]
*Simplicidiella nigra*	CBMAI 1939	-	KU216313	-	KU216291	KT833147	-	KU216338 *	-	[171]
*Sparticola junci*	MFLUCC 15-0030	NG_061235	KU721765	-	-	NR_154428	KU727900	KU727898	-	[146]
*Staninwardia suttonii*	CPC 13055	-	KF901874	KF903517	KF902693	KF901552	KF902392	KF903270 *	-	[49]
*Staurosphaeria lycii*	MFLUCC 17-0210	MF434372	MF434284	-	-	MF434196	-	MF434458	-	[134]
*Staurosphaeria lycii*	MFLUCC 17-0211	MF434373	MF434285	-	-	MF434197	-	MF434459	-	[134]
*Stenella araguata*	FMC 245	-	KF902168	-	-	-	KF902393	-	-	[49]
*Suberoteratosphaeria pseudosuberosa*	CPC 12085	-	KF902144	KF903508	-	KF901786	-	KF903275 *	-	[49]
*Suberoteratosphaeria xenosuberosa*	CPC 13093	-	KF901879	KF903584	-	KF901557	KF902402	KF903280 *	-	[49]
*Teichospora mariae*	C136	-	KU601581	-	-	KU601581	KU601595	KU601611	-	[184]
*Teichospora rubriostiolata*	TR 7	-	KU601590	-	-	KU601590	KU601599	KU601609	-	[184]
*Teichospora thailandica*	MFLUCC 17-2093	MT226708	MT214597	-	-	MT310641	MT394708	MT394653	-	[167]
*Teichospora trabicola*	C 134	-	KU601591	-	-	KU601591	KU601600	KU601601	-	[184]
*Teratoramularia infinita*	CBS 141104	-	KX287249	KX287828	KX289125	KX287545	KX288710	KX288107 *	-	[125]
*Teratoramularia rumicicola*	CBS 141106	-	KX287255	-	-	KX287550	KX288716	KX288113 *	-	[125]
*Teratosphaeria aurantia*	MUCC 668	-	KF901884	KF903578	KF902700	KF901561	KF902409	KF903284 *	-	[49]
*Teratosphaeria blakelyi*	CPC 12837	-	KF901888	KF903518	KF902704	KF901565	KF902413	KF903288 *	-	[49]
*Teratosphaeria destructans*	CPC 1368	-	KF901898	KF903447	KF902716	KF901574	KF902427	KF903301 *	-	[49]
*Teratosphaeria fimbriata*	CPC 13324	-	KF901901	KF903529	KF902720	KF901577	KF902430	KF903306 *	-	[49]
*Teratosphaeria gauchensis*	CMW 17331	-	KF902148	KF903521	KF902729	KF901790	KF902439	KF903315 *	-	[49]
*Teratosphaeria mareebensis*	CPC 17272	-	KF901906	KF903581	KF902734	KF901582	KF902444	KF903320 *	-	[49]
*Teratosphaeria pseudocryptica*	CPC 11267	-	KF902032	KF903598	KF902760	KF901687	KF902472	KF903348 *	-	[49]
*Teratosphaeriaceae* sp.	CPC 13680	-	KF901921	KF903657	KF902765	KF901597	KF902477	KF903353 *	-	[49]
*Teratosphaeriaceae* sp.	CCFEE 5569	-	KF310015	-	-	-	KF310071	-	-	[139]
*Teratosphaericola pseudoafricana*	CPC 1231	-	KF902045	KF903435	KF902782	KF901699	KF902499	KF903370 *	-	[49]
*Teratosphaericola pseudoafricana*	CPC 1230	-	KF902084	KF903473	KF902783	KF901737	KF902500	KF903371 *	-	[49]
*Teratosphaeriopsis pseudoafricana*	CPC 1261	-	KF902085	KF903436	KF902784	KF901738	KF902501	KF903372 *	-	[49]
*Vaginatispora amygdali*	KT 2248	LC312495	LC312553	-	-	LC312524	LC312611	LC312582	-	[140]
*Vaginatispora appendiculata*	MFLUCC 16-0314	KU743219	KU743218	-	-	KU743217	-	KU743220	-	[185]
*Vaginatispora armatispora*	MFLUCC 18-0247	MK085058	MK085060	-	-	MK085056	MK087669	MK087658	-	[146]
*Vaginatispora nypae*	MFLUCC 18-1543	NG_065779	NG_066313	-	-	NR_163340	MK434877	MK360091	-	[127]
*Vaginatispora scabrispora*	KT 2443	LC312496	LC312554	-	-	LC312525	LC312612	LC312583	-	[140]
*Westerdykella ornata*	CBS 379.55	GU296208	GU301880	-	-	AY943045	-	GU349021	-	[142]
*Xenopenidiella inflata*	CBMAI 1945	-	KU216337	-	KU216312	KT833171	-	KU216359 *	-	[171]
*Xenopenidiella tarda*	CBMAI 1940	-	KU216326	-	KU216303	KT833160	-	KU216351 *	-	[171]
*Xenophacidiella pseudocatenata*	CPC 18472	-	KF902092	-	-	-	KF902508	-	-	[49]
*Xenopyrenochaetopsis pratorum*	CBS 445.81	NG_062792	NG_057858	-	-	NR_111623	KT389671	-	KT389846	[186]

GenBank accession numbers with * are resulting from EF1-728F and EF-2 primers and – means missing data or not used in the phylogenetic analyses. The newly generated sequences are indicated in bold.

**Table 4:** Maximum-likelihood (ML) and Bayesian (BI) analyses results for each sequenced dataset.

Analyses		Teratosphaeriaceae	*Rhytidhysteron*	Lophiostomataceae	*Parapyrenochaeta*
Number of Taxa		106	34	106	37
Gene regions		LSU, ITS, *rpb*2, *act*, *cal* and *tef*1	SSU, LSU, ITS and *tef*1	SSU, LSU, ITS, *tef*1 and *rpb*2	LSU, SSU, ITS, *rpb*2, *tef*1 and *btub*
Number of character positions (including gaps)		3517	3667	4649	5510
ML optimization likelihood value		−50604.86449	−10388.988691	−42280.12689	−27947.901235
Distinct alignment patterns in the matrix		1973	739	2082	1710
Number of undetermined characters or gaps (%)		48.76%	30.69%	27.07%	38.18%
Estimated base frequencies	A	0.23693	0.241388	0.24893	0.245506
	C	0.26813	0.244326	0.24732	0.244909
	G	0.283733	0.277859	0.267917	0.265204
	T	0.211207	0.236427	0.235833	0.244381
Substitution rates	AC	1.498833	1.533268	1.549406	1.619926
	AG	2.784366	2.507774	4.37387	4.391077
	AT	1.662835	1.340621	1.462392	1.995039
	CG	1.129905	1.029121	1.453674	1.225921
	CT	6.210175	6.529612	8.808274	8.980921
	GT	1.0	1.0	1.0	1.0
Proportion of invariable sites (I)		0.416989	0.610823	0.453545	0.55191
Gamma distribution shape parameter (α)		0.626612	0.475911	0.51454	0.443538
Number of generated trees in BI		29861	3451	9001	951
Number of trees sampled in BI after 25% were discarded as burn-in		22396	2589	6751	714
Final split frequency		0.009999	0.009261	0.009977	0.007923
The total of unique site patterns		1974	740	2084	1711

## Data Availability

The datasets generated for this study can be found in the NCBI GenBank, MycoBank and TreeBASE.
